# Bystander CD8^+^ conventional memory versus virtual memory T cells in the initial days post-*Trypanosoma cruzi* infection

**DOI:** 10.3389/fimmu.2025.1674964

**Published:** 2025-12-08

**Authors:** Maria Estefania Viano, Ruth Eliana Baigorri, Gastón Bergero, Maria Pilar Aoki, Nicolas Leonel Lidon, Maria Guadalupe Teixeira, Melisa Rocio Herrera, Claudia Cristina Motran, Fabio Marcelo Cerban, Cinthia Carolina Stempin, Maria Cecilia Rodriguez-Galan

**Affiliations:** Inmunología, CIBICI-CONICET, Departamento de Bioquímica Clínica, Facultad de Ciencias Químicas, Universidad Nacional de Córdoba, Córdoba, Argentina

**Keywords:** bystander, Ag-independent conventional CD8+ T cells, TMEM, virtual memory CD8+ T cells, TVM, *Trypanosoma cruzi*, IFNγ, NKG2D

## Abstract

**Background:**

Bystander activation has primarily focused on conventional antigen-specific T cells (T_MEM_) and other innate immune cell types. However, the role of virtual memory T cells (T_VM_) has been largely overlooked, despite their numerical superiority and highly cytotoxic phenotype. Bystander activation is particularly relevant in infections caused by intracellular pathogens. In this study, we aimed to compare the bystander activation potential of T_VM_ cells versus T_MEM_ cells during the early days following *T. cruzi* infection.

**Methodology/Principal Findings:**

Our results demonstrate that T_VM_ and T_MEM_ cells, evaluated by flow cytometry, are present but do not undergo significant changes in frequency during the first four days post-infection (p.i.). In an *in vitro* co-culture system, T_VM_ or T_MEM_ cells pre-incubated with IL-12 and IL-18 (effector cells) were cultured with *T. cruzi*-infected enriched peritoneal macrophages (Tc-PM, target cells). Immunofluorescence assays revealed that both T_VM_ and T_MEM_ cells exhibit a highly efficient capacity to kill the parasite and induce degranulation, in contrast to naïve T cells (T_N_), which showed almost no cytotoxic activity. Furthermore, intracellular flow cytometry assays confirmed that both T_VM_ and T_MEM_ cells produce substantial amounts of IFNγ up to 4 days p.i. when stimulated *in vitro* with IL-12 and IL-18, whereas T_N_ cells fail to produce this cytokine. Accordingly, T_VM_ and T_MEM_ cells exert their cytotoxic effects via IFNγ production, rather than NKG2D, which subsequently activates reactive oxygen species (ROS) and Nitric Oxide (NO) pathways in Tc-PM. Additionally, we demonstrate that in T_VM_ cells, IFNγ signaling occurs through STAT1 in Tc-PM. Finally, analysis of human T_VM_ cells within PBMCs, revealed increased expression of the functional marker granzymes in Chagas disease patients compared to healthy controls.

**Conclusions/Significance:**

These results challenge the view that only T_MEM_ cells dominate early infection control. The equivalency of T_VM_ and T_MEM_ cells in parasite clearance suggests T_VM_ cells are valuable innate-like contributors, providing rapid protection. Their numerical prevalence in unprimed individuals indicates T_VM_ cells may be an underestimated component of early immunity.

## Introduction

Memory CD8^+^ T cells (T_MEM_) promptly mount antigen (Ag)-specific immune responses during pathogen reencounters. However, T_MEM_ cells also react to inflammatory cues without an activating TCR signal, a phenomenon known as bystander activation (1–4).

Although bystander activation was initially described over two decades ago, its physiological relevance and consequences have only recently become more evident. The direct functional outcomes of TCR-mediated and bystander-mediated T cells activation appear remarkably similar. In both cases, they can include T cell proliferation (5), cytokine expression (1, 6), and direct target cytolysis (7, 8). Importantly, numerous studies have documented the benefit of effector responses by bystander-activated T cells in multiple animal models of infection, including *L. monocytogenes* (1, 7), *Influenza A virus* (9), *Y. pseudotuberculosis* (10), *murine gamma herpes virus 4* (11), and *S. aureus pneumonia* (12).

One potential reason for the limited evidence of bystander protection of memory T cells from pathogens might be due to redundancy with similar responses from other cell types, such as unconventional lymphocyte populations as ILCs, γδT cells, NK cells and NKT cells (13, 14). In this context, the role of antigen-inexperienced virtual memory CD8^+^ T cells (T_VM_) gains significant relevance due to their abundance in secondary lymphoid organs (SLO) and circulation. T_VM_ cells not only share multiple phenotypic and functional characteristics with antigen-specific memory T cells (T_MEM_) but also comprise approximately 50% and 80% of the total CD8^+^ CD44^hi^ population in the spleen and lymph nodes, respectively (15–17). Consequently, the potential bystander response of abundant T_VM_ cells could vastly surpass the bystander protection mediated by T_MEM_ cells and other innate cells that participate early in immune responses against pathogens in mice. Furthermore, feral mice kept in captivity displayed an enlarged T_VM_ compartment, that highly exceeds the number of Ag-experienced T_MEM_ cells (18).

Bystander activation relies on the presence of inflammatory cues and occurs swiftly and transiently in the early stages of an immune response, beginning as early as 24 hours post-infection and lasting for 3 to 5 days (1, 7, 19). The following cytokines in combination are sufficient to trigger bystander activation: type I IFN, IL-12, IL-15, and IL-18 (1, 6, 19, 20). For example, without the involvement of specific cognate antigenic signals, the proinflammatory cytokines IL-12 and IL-18 induce the differentiation of memory CD8^+^ T cells into effector cells, characterized by rapid activation and robust IFNγ production (1, 21). Moreover, these IFNγ-producing memory CD8^+^ T cells can provide an early protective response against intracellular pathogens in an Ag-nonspecific manner (1). Additionally, T_VM_ cells also undergo bystander activation due to their high expression levels of IL-12 and IL-18 receptors, producing large amounts of IFNγ upon stimulation with IL-12/IL-18 *ex vivo (*22, 23). In turn, IFNγ has been shown to activate microbicidal effector programs in macrophages and other antigen-presenting cells (APCs), including the production of reactive oxygen species (ROS) and nitric oxide (NO), increased phagocytosis, and the upregulation of antigen presentation and costimulatory molecules (24, 25).

Besides the multifaceted functions of IFNγ, IL-15 produced early after infection can activate memory CD8^+^ T cells, upregulating the expression of NK receptors such as NKG2D (7, 26, 27). This, in turn, can mediate direct cell killing without the need for TCR stimulation (27). The immunoreceptor NKG2D interacts with a range of stress-induced NKG2D ligands (NKG2DLs), which act as general indicators of infection, stress, or cellular transformation (26, 27). NKG2D facilitates the elimination of cells expressing NKG2DLs. Although NKG2D levels in T_VM_ cells are lower than in T_MEM_ cells (28–30), its expression is significantly higher than in naïve T cells (23). However, the NKG2D effector mechanism has not yet been investigated in T_VM_ cells.

Considering all the evidence presented, it remains unclear why the importance of T_VM_ cells has not been investigated during the process of bystander activation, especially in comparison to the role of T_MEM_ cells, which have been extensively studied and given greater prominence (15, 16).

Therefore, in the present study, we focus on comparatively evaluating the cytotoxic role of both cell populations during the initial days of *Trypanosoma cruzi* infection, an intracellular parasite in which our group has reported a protective role for T_VM_ cells (31). Our data demonstrates that T_VM_ cells function as potent memory cells, responding as robustly and efficiently as pre-existing T_MEM_ cells in a bystander manner during the early stages of *T. cruzi* infection. Upon stimulation with IL-12 and IL-18, these cells rapidly produce IFNγ, which subsequently signals within *T. cruzi*-infected macrophages. This activation induces the production of reactive oxygen species (ROS) and nitric oxide (NO), leading to the effective elimination of the pathogen.

Interestingly, T_VM_ cells also exist in humans where, the expression of Killer-cell Immunoglobulin-like Receptors (KIRs) or Natural Killer Group 2, member A (NKG2A) on CD8^+^ T cells is considered a consensus marker of T_VM_ cells (32). Several characteristics of KIR or NKG2A^+^ CD8+ T cells in humans are similar to their murine counterparts, including the exertion of innate-like effector functions via IL-12/IL-18 or IL-15 stimulation without TCR stimulation (32).

Importantly, bystander activation of non-Ag-specific T_MEM_ cells has also been observed in human cohorts during systemic viral infections, including primary HIV (33), primary EBV (34), and during acute dengue virus infections (35). However, the role of bystander activation in human memory T cells appears to be a two-edged sword. While some studies indicate potential benefits for disease control, others associate it with immunopathological and autoimmune processes (16).

In the present study, we demonstrate that T_VM_ cells in the blood of chronically infected Chagas patients exhibit higher expression of proliferative (Ki67) and functional (perforin and granzymes) markers compared to healthy individuals. Whether these characteristics contribute to protection or trigger pathology, such as cardiomyopathy in this infection, remains to be evaluated.

Our data presented in this work highlights the evidence that T_VM_ cells should be considered a major contributor during bystander activation, demonstrating not only functional capacities comparable to T_MEM_ cells but also outnumbering any other innate or antigen-independent immune response early in the infectious process. Moreover, bystander activation is a critical component of the early and potentially chronic immune response to *T. cruzi* infection, which could lead to either enhanced protection or the development of pathology during chronic infection.

## Materials and methods

### Ethics statement

All experimental procedures were approved by the Institutional Animal Care and Use Committee (IACUC) of the Facultad de Ciencias Químicas, Universidad Nacional de Córdoba (authorization no. RD-2022-1042-E-UNC-DEC#FCQ). This committee adheres to the guidelines outlined in the “Guide to the Care and Use of Experimental Animals” (Canadian Council on Animal Care, 1993) and the “Institutional Animal Care and Use Committee Guidebook” (ARENA/OLAW IACUC Guidebook, National Institutes of Health, 2002). Additionally, our animal facility holds an NIH animal welfare assurance (assurance number A5802-01, OLAW, NIH, USA).

### Mice

Experiments were conducted using male and female wild-type C57BL/6 (B6), OT-I (RAG-sufficient, B6 background) and IFNαR KO mice, aged between 6 and 10 weeks, housed under specific pathogen-free conditions. Type I interferon receptor-deficient mice (IFNαR KO, Ifnar1tm1Ag) were provided by the Institute Pasteur.

Following completion of the experimental procedure, animals were humanely euthanized via cervical dislocation, in accordance with institutional ethical guidelines.

### *In vivo Candica albicans* and *Trypanosoma cruzi* infection

*Trypanosoma cruzi* (Tulahuen strain) trypomastigotes were propagated through serial passages in wild-type BALB/c mice. For experimental procedures, B6 mice were infected intraperitoneally (i.p.) with 5 x 10^5^*T. cruzi* trypomastigotes suspended in PBS as previously reported by our laboratory (31, 36). Mice were euthanized on days 2, day 4 or day 14 post-infection.

Yeast cells of *C. albicans* (SC5314 strain) were grown on Sabouraud glucose agar slopes at 28˚C and maintained by weekly subculture. B6 mice were i.p. injected with 3 x 10^7^ viable yeast diluted in PBS as previously described in our laboratory (31, 36). Mice were euthanized 2 days after the infection.

### Enriched-peritoneal macrophages

C57BL/6 mice were intraperitoneally infected as previously described; bulk peritoneal cells were collected through multiple peritoneal lavages using PBS supplemented with 3% FBS (Natocor, Argentina). Non-infected mice were processed in parallel to serve as controls. For *in vitro* experiments, bulk peritoneal cells were seeded in a 24- or 96- well plates with complete medium and allowed to adhere for 3 hours. Following adhesion, wells were washed three times to eliminate non-adherent cells (37).

### *In vitro T. cruzi* infection and co-culture assays

Blood-derived *T. cruzi* (Tulahuen strain) trypomastigotes were used to infect Vero cell monolayers. After a 7-day incubation, supernatants with the parasite were collected and stored at -80 °C. For *in vitro* infection of enriched-PM, bulk peritoneal cells were obtained as previously described and infected with *T. cruzi* at a 1:5 ratio (cells:parasites). The following day, infected enriched-PM (Tc-PM, targets) were thoroughly washed to eliminate any excess parasites and co-cultured with bulk splenocytes or sorted T_N_, T_VM_ or T_MEM_ cells from WT C57BL/6 mice pre-treated ON with IL-12 (10 ng/mL) and IL-18 (100 ng/mL) (effectors), using a 1:3 ratio (targets: effectors). Cultures were maintained at 37 °C with 5% CO_2_.

### *In vitro* cytotoxic assays

For *in vitro* cytotoxicity assays, using or not neutralizing antibodies, Tc-PM were obtained as previously described. Enriched-PM were co-cultured with T_N_, T_VM_, or T_MEM_ cells, previously purified by cell sorting (see Cell Sorting section) and pre-stimulated for 16 h with IL-12 (10 ng/mL) and IL-18 (100 ng/mL). For the co-culture experiments, a 1:1 target-to-effector cell ratio was used, with 1 x 10^5^ cells of each type. Neutralizing anti-NKG2D (20 μg/mL) or anti-IFNγ (20 μg/mL) or the corresponding isotype control antibodies were added to the co-cultures. After 48 h of co-culture, flow cytometry was used to assess the frequency of dead target cells (F4/80^+^CD11b^+^Zombie^+^) and the expression of the degranulation marker CD107a in effector cells. In addition, the number of infected cells was evaluated by immunofluorescence. The number of viable trypomastigotes was determined in 72 h co-culture supernatants by counting motile forms observed under an optical microscope at 40x magnification.

### Immunofluorescence staining

The presence of parasites inside Tc-PM was determined by visualizing intracellular amastigotes using immunofluorescence as previously described by our laboratory (38, 39). Briefly, coverslips placed in the co-cultures were collected 48 h post-*Trypanosoma cruzi* infection for staining. Cells were washed with PBS and fixed with 4% paraformaldehyde for 40 minutes. Following fixation, coverslips were washed again with PBS and permeabilized with 1% Triton X-100 for 15 minutes. After another PBS wash, cells were blocked with 1% BSA for 15 minutes. Samples were then incubated with serum from a Chagas disease patient which contains anti-*T. cruzi* immunoglobulins (Igs), followed by incubation with FITC-conjugated anti-human IgG. For nuclear staining, coverslips were incubated with 4′,6-diamidino-2-phenylindole (DAPI), then washed with PBS and mounted using FluorSave mounting medium overnight.

### Conventional flow cytometry and cell sorting

Phenotypic analysis of splenocytes was performed by *ex vivo* flow cytometry at different days post-infection. Spleens were harvested and mechanically disrupted with a disposable mesh (FiltraBags). Splenocyte suspensions were depleted of red cells by treatment with ACK lysis buffer before staining. The samples were first washed with PBS and stained with Zombie Aqua Fixable Viability Kit (BioLegend; Cat# 423102) for 15 minutes at room temperature to exclude dead cells. Expression of different surface markers was assessed by staining with appropriate combinations of monoclonal antibodies (mAbs) for 30 minutes at 4 °C, AF700-CD8 (clone: 53-6.7, Cat#: 100729, BioLegend), FITC-CD44 (clone: IM7, Cat#: 11-0441-82, eBioscience), PECy7-CD49d (clone: R1-2, Cat#: 103618, BioLegend). Cells were washed twice with PBS and acquired on a BD LSR Fortessa X-20 cytometer (BD Biosciences).

To evaluate intracellular IFNγ expression, cells were stimulated overnight with IL-12 (10 ng/mL) and IL-18 (100 ng/mL) and 5 μg/mL of both Brefeldin A and Monensin (Sigma) were added during the last for 4 h. Cells were then stained for surface markers, washed, and fixed with Cytofix/Cytoperm buffer (BD Pharmingen) for 30 minutes at 4 °C. Following fixation, cells were washed with Perm Wash buffer (BD Pharmingen) and incubated with PerCP-Cy5 anti-mouse IFNγ antibody (clone: XMG1.2, Cat#: 560660, BD Pharmingen) or an isotype-matched control antibody (clone: MOPC-21, Cat#: 552834, BD Pharmingen) for 30 minutes at 4 °C. After two final washes, samples were analyzed using flow cytometry.

For cell sorting, splenocytes were isolated from WT C57BL/6 mice as previously described and stained with Zombie dye, CD8, CD44, and CD49 antibodies. Using a BD FACSAria IIu Cell sorter, T_N_ (CD8^+^ CD44^-^ CD49d^-^), T_VM_ (CD8^+^ CD44^+^ CD49d^-^), and T_MEM_ (CD8^+^ CD44^+^ CD49d^+^) subsets were identified within Zombie^-^ viable cells. The sorting process yielded populations with a high purity of 97–99%, ensuring accurate downstream analyses.

To assess RAE expression, PM were obtained from control and *T. cruzi*-infected mice at days 2, 4, and 14 post-infection and RAE expression was determined in the F4/80^+^ CD11b^+^ population. FITC-F4/80 (clone:BM8, Cat#: 123107, Biolegend), PE-RAE (clone: 186107, Cat#: FAB17582P, R&D systems), APC-CD11b (Clone:M1/70, Cat#: 553312, BD).

For the assessment of pSTAT1, PM were isolated from IFNαR-KO mice. PM were stained using PECy7-F4/80 (Clone: BM8, Cat# 123107) and APC-CD11b (Clone: M1/70, Cat# 553312, BD), then plated and infected with *T. cruzi* as previously described.

In parallel, for the STAT1 experiment, T_VM_ cells from control C57BL/6 mice were sorted based on CD8, CD44 and CD49d markers as reported in this section, and stimulated overnight with IL-12 (10 ng/mL) and IL-18 (100 ng/mL), with or without a neutralizing anti-IFNγ antibody (20 μg/mL). Following stimulation, the supernatants from these cultures were added to the previously Tc-PM.

Tc-PM were promptly collected within 10 minutes post-supernatant addition and immediately fixed (10 min, 37 °C) using BD Cytofix™ Fixation Buffer (Cat#: 554655). They were then permeabilized on ice for 30 minutes with BD Phosflow™ Perm Buffer III (Cat#: 558050). After two washes in BD Pharmingen™ Stain Buffer (Cat#: 554656), the cells were stained with PE-anti-STAT1 (pY701) antibody (Cat#: 562069). Finally, flow cytometric analysis was performed using a BD FACS-Fortessa.

ROS production in PM was assessed at 48 h, and NO production at 72 h post-supernatant addition of sorted T_VM_ or T_MEM_ cells from WT mice, stimulated overnight with IL-12 (10 ng/mL) and IL-18 (100 ng/mL), with or without a neutralizing anti-IFNγ antibody (20 μg/mL). As positive control, PM were incubated with recombinant IFNγ (rIFNγ) (10ng/ml).

For cytoplasmic ROS analysis, cells were previously stained for surface markers (PECy7-F4/80 Clone: BM8, Cat# 123107; APC-CD11b Clone:M1/70, Cat#: 553312, BD), washed and then incubated with 20 µM H2DCFDA (Cat #:D399, Invitrogen) probe in PBS for 30 min at room temperature. For NO detection, cells were incubated with 20 μM DAF-FM probe (4-Amino-5-Methylamino-2’,7’-Difluorofluorescein Diacetate, Cat #: D23844) for 30 min at 37 °C. Then, cells were washed and stained with Zombie Aqua Fixable Viability Kit (Cat# 423102, Biolegend) for 15 minutes at room temperature to exclude dead cells. Subsequently, cells were washed with FACS buffer and analyzed on a BD LSR Fortessa X-20 cytometer.

### Human peripheral blood mononuclear cells from Chagas patients

PBMCs were obtained from adult volunteers recruited at Hospital Nuestra Señora de la Misericordia and the Central Laboratory of Córdoba, Argentina. PBMCs were isolated from venous blood samples using Ficoll-Hypaque PLUS (GE Healthcare Bioscience) density gradient centrifugation. The study included 7 patients diagnosed with asymptomatic chronic Chagas disease (5 males and 2 females; age range: 45–65 years; median: 50 years), whose infection was confirmed by positive results in both indirect hemagglutination and ELISA assays. All infected individuals underwent clinical evaluation, including electrocardiography (ECG) and chest radiography, with no pathological findings reported. The control group (healthy donors=HD) comprised 7 seronegative adults (3 males and 4 females; age range: 28–52 years; median: 42 years). This study was reviewed and approved by the Comité Institucional de Ética de la Investigación en Salud del Adulto, Ministerio de Salud de la Provincia de Córdoba (Act 331/2018). All studies were conducted in accordance with the principles outlined in the Declaration of Helsinki. Signed informed consent documents were obtained from each donor included in the study.

Approximately 15–30 ml of peripheral blood was drawn from each individual. PBMCs were isolated through density gradient centrifugation using Ficoll-Hypaque PLUS (GE Healthcare Bioscience) and then frozen in SBF containing 10% DMSO and stored in liquid nitrogen until use.

PBMCs were stimulated with PMA (50 ng/mL) and Ionomycin (500 ng/mL) for 4 h with 5 μg/mL of both Brefeldin A and Monensin (Sigma) added during the last 3 hours. Cells were first stained with Zombie Aqua Fixable Viability Kit (Cat# 423102, Biolegend) for 15 minutes at room temperature to exclude dead cells. Subsequently, cells were washed with FACS buffer and incubated with the combination of anti-human monoclonal antibodies listed below for 20 min in the dark at 4 °C: PECy7-CD8, Clone: RPA-T8, Cat#: 301012, Biolegend; PE-DazzleTCRVα24, Clone: 6B11, Cat#: 342920, Biolegend; BV785-TCRVα7.2, Clone: 3C10, Cat#: 351722, Biolegend; BV421-TCRαβ, Clone: IP26, Cat#: 306722, Biolegend; PE-CD158e/k (KIR2DL1/DL2), Clone: 5.133, Miltenyi Biotec; PE-KIR2D, Clone: NKVFS1, Miltenyi Biotec; AF700-CD159a (NKG2A), Clone: S19004C, Cat#: 375120, Biolegend. Then, cells were washed, and fixed with Cytofix/Cytoperm buffer (BD Pharmingen) for 30 minutes at 4 °C. Following fixation, cells were washed with Perm/Wash buffer (BD Pharmingen) and incubated with EF660-Eomes, Clone: WD1928, Cat#:50-4877-42, eBioscience; APCCy7-perforin, Clone: dG9, Cat#: 308128, Biolegend; PECy5-granzyme B, Clone: QA16A02, Cat#: 372226, Biolegend; FITC-IFNγ, Clone: 4S.B3, Cat#: 502507, Biolegend; BV605-Ki67, Clone: Ki67, Cat#: 350522, Biolegend and PCPCy5.5-Helios, Clone: 22F6, Cat#: 137230, Biolegend; for 30 minutes at 4 °C. After two final washes, samples were analyzed on a BD LSR Fortessa X-20 cytometer.

### Statistical analysis

Statistical analysis of the data was performed using GraphPad Prism software (version 9.00). In all cases, data obtained from different groups were subjected to the ROUT test (Q = 1) and Grubbs’ test (α=0.05) for outlier detection. Subsequently, the following statistical tests were applied: one- or two-tailed ANOVA, as appropriate for each experimental condition, and Student’s t-test. Simple regression analysis was performed for experiments with human samples. Results were presented as mean ± standard error of the mean (SEM).

## Results

Bystander activation of T cells operates during the initial days post-infection and has been reported to play a protective role in various infectious contexts until the antigen-specific immune response takes place (1–4). During this time, a powerful arm of the immune system, such as the role of virtual memory T cells, has been overlooked. Moreover, the role of bystander activation of these types of T cells has not been explored during parasitic infections.

**Figure 1:**
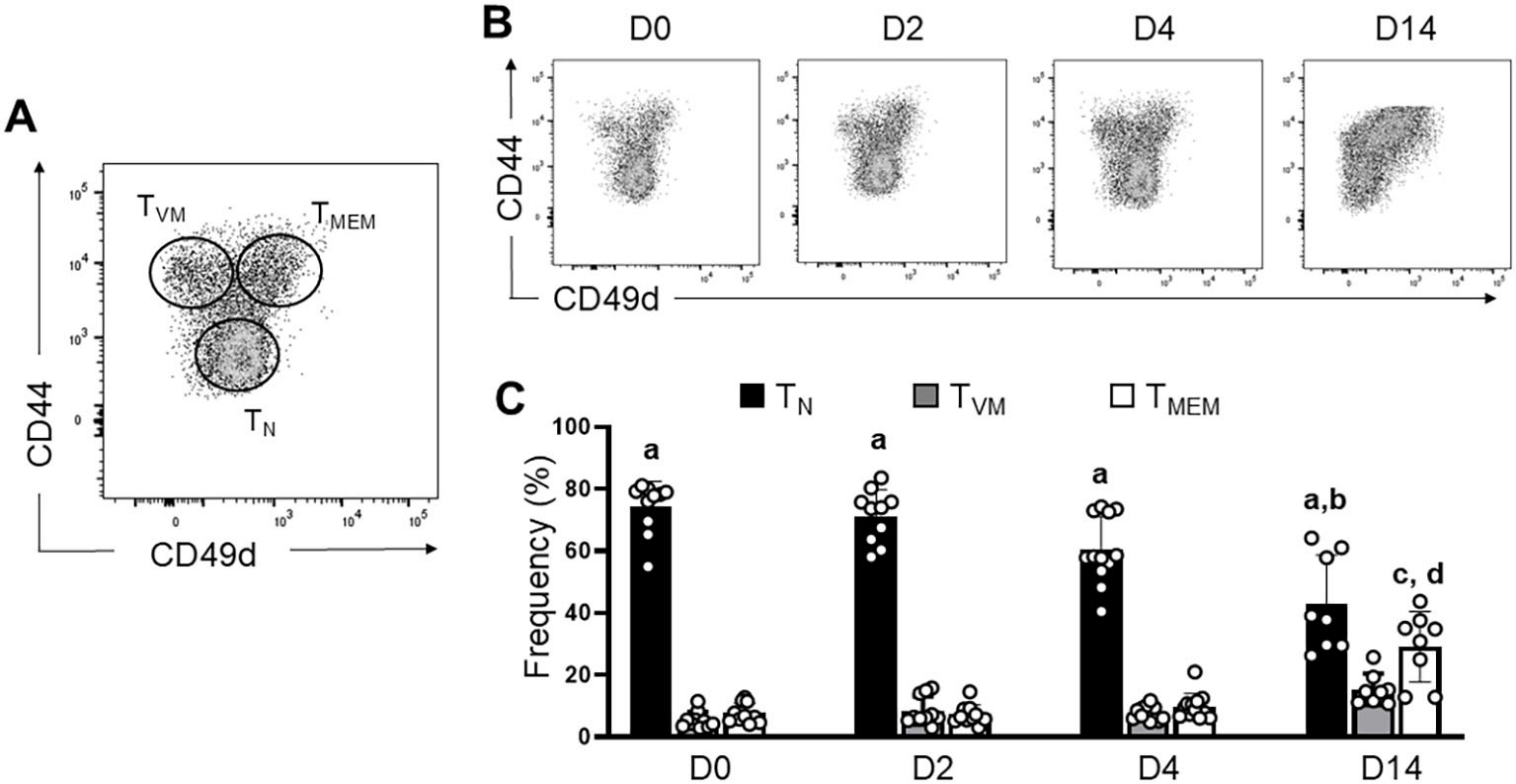
Early dynamics of memory T cells during *T. cruzi* infection. **(A)** Representative dotplot of different subsets of splenic CD8^+^ T cells. Naïve T cells (T_N_) are defined as CD44^lo^ CD49d^lo^, whereas memory T cells (CD44^hi^) are subdivided into Ag-specific conventional T_MEM_ (CD49d^hi^) and T_VM_ (CD49d^lo^) cells. **(B, C)** Comparative analysis of T_MEM_ and T_VM_ cells frequencies at different time points post-infection. Data are the pool of three independent experiments with 3–5 mice per group. Bar graph data are shown as the mean ± SEM. Statistical analysis was performed using two-way ANOVA followed by Tukey’s post-test. a, T_N_ D0-D14 versus T_VM_ D0-D14 and T_MEM_ D0–14 p=0.02. b, T_N_ D0-D4 versus T_N_ D14 p<0.001. c, T_MEM_ D14 versus T_MEM_ D0-D4 p<0.0001. d, T_MEM_ D14 versus T_VM_ D14 p=0.03.

In this comparative study between pre-existing T_MEM_ cells and T_VM_ cells, we first evaluated their frequency in the initial days post-*T. cruzi* infection (p.i.) and compared it with Day 14 p.i. (D14), when Ag-specific effector T cells start to gain importance (**Figure 1**). T_MEM_ and T_VM_ cells are both memory T cells (CD44^hi^) that can be distinguished by CD49d expression. T_MEM_ cells upregulate CD49d after TCR engagement, as previously reported (15). Conversely, T_VM_ cells do not primarily operate through TCR recognition and thus remain CD49d^lo^ (15). These two types of memory cells can also be differentiated in the same dot plot from T naïve (T_N_) cells, which are CD44^lo^ CD49d^lo^ (**Figures 1A, B** and gate strategy at **Supplementary Figure 1**). As seen in **Figures 1C**, frequency of T_N_ cells is significantly higher than T_VM_ and T_MEM_ cells throughout the entire study period, although its frequency shows a decrease at D14 compared to previous days, due to the expected expansion of Ag-specific effector/memory cells against the pathogens (T_MEM_ D14) as demonstrated by our laboratory (31). Consequently, T_MEM_ cells are increased at D14 compared to earlier days.

Interestingly, T_VM_ cells show a tendency to increase at D14 compared to previous days, but this difference is not significant, suggesting that T_VM_ frequency does not appear to rise during the onset of the adaptive immune response (D14). Another noteworthy point is that in the early days post-infection, the frequency of T_VM_ vs T_MEM_ cells is similar; however, by D14, T_MEM_ cells increased significantly compared to T_VM_ cells on the same day.

This initial observation leads us to consider that T_VM_ cells might contribute similarly to the bystander response exhibited by T_MEM_ cells during the early stages of *T. cruzi* infection. This concept will be evaluated throughout the course of this study.

**Figure 2:**
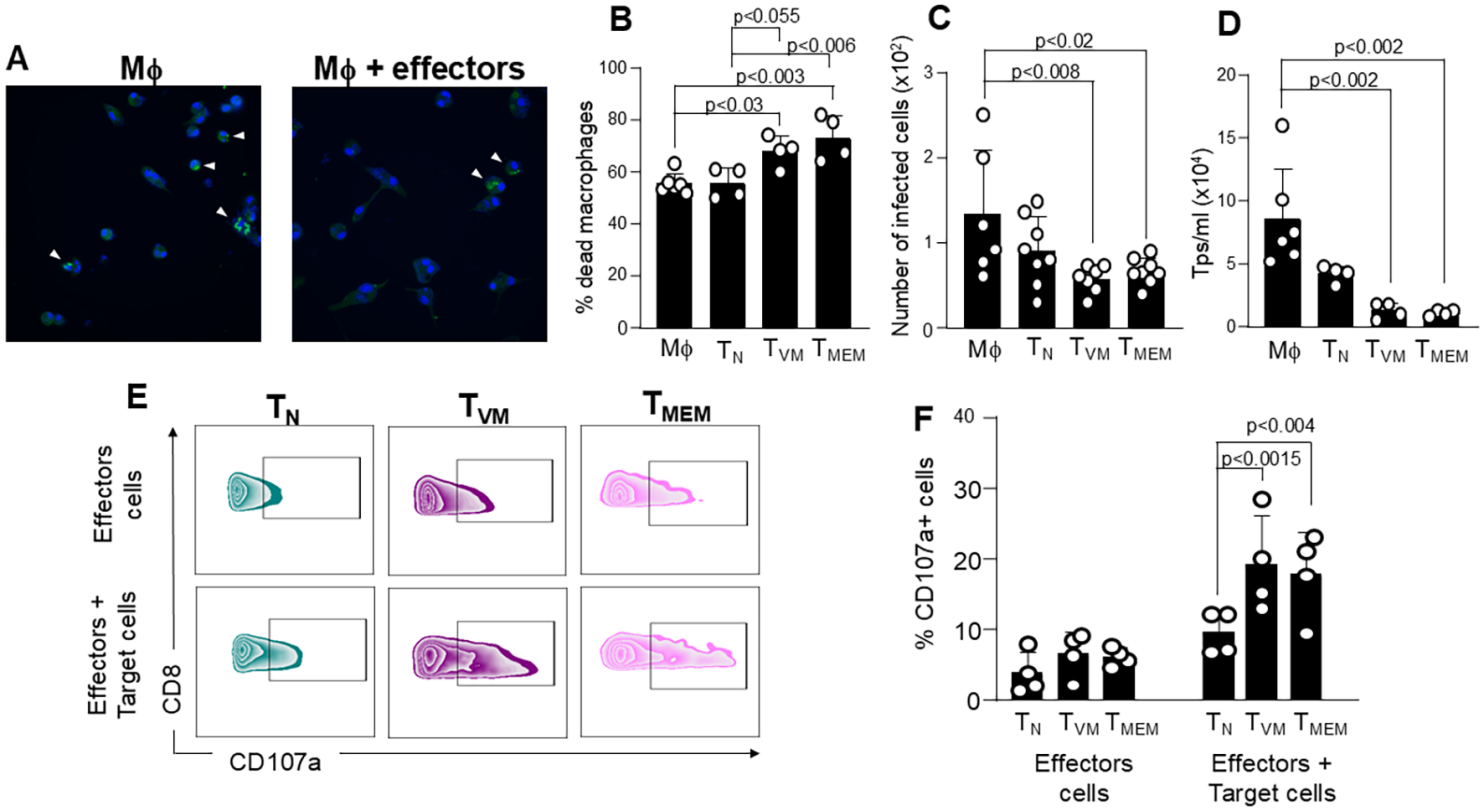
Cytotoxic activity of T_MEM_ and T_VM_ cells against enriched *T. cruzi*-infected macrophages. **(A-F)***In vitro* cytotoxicity assays. Effector T cell subsets (T_N_, T_VM_, and T_MEM_) were preincubated overnight with IL-12 and IL-18 to mimic *in vivo* conditions, and then co-cultured with enriched Tc-PM. **(A)** Representative photograph of Tc-PM alone (Mϕ, left) or after co-cultured with T_N_, T_VM_ or T_MEM_ cells (effectors, right). Nuclei are stained with DAPI and parasites appear in green (FITC). **(B)** Bar graph shows the percentage of dead macrophages evaluated by aqua zombie dye in the F4/80^+^ CD11b^+^ population by flow cytometry. **(C)** Number of intracellular infected target cells, evaluated by immunofluorescence staining 48 h post-co-culture. **(D)** Number of Trypomastigotes (Tps) in the culture supernatants, measured 72 h after co-culture. **(E, F)** Flow cytometry analysis of CD107a expression, a marker of degranulation, in effector cells co-cultured with Tc-PM target cells. Data are representative of 2 independent experiments. Bar graph data are shown as the mean ± SEM. Statistical analysis was performed using one-way ANOVA followed by Tukey’s post-test.

*Trypanosoma cruzi* infects various types of cells; however, it exhibits a particular tropism for macrophages (25). These macrophages become highly activated in the initial days post-infection, playing a crucial role in controlling the parasite (25). They produce significant amounts of IL-12 and IL-18, which can trigger the bystander activation of T_MEM_ and T_VM_ cells, as these cells constitutively express receptors for both cytokines (1, 21–23, 40). The comparative *in vivo* role of T_MEM_ and T_VM_ cells is challenging to study because it would require the elimination of cells based on CD8, CD44 or CD49d expression. This is impractical since these markers are widely expressed by most leukocytes, including DCs, NK cells, NKT cells and γδT cells, which also contribute to the innate immune response (41). Therefore, in our studies, we conducted a comparative and simultaneous evaluation of the killing capacity of T_MEM_, T_VM_, and T_N_ cells (effectors) on *T. cruzi*-infected macrophages (targets) using an *in vitro* approach. To mimic the *in vivo* situation, we pre-incubated the effector cells overnight with IL-12 and IL-18 and then co-cultured them with previously enriched *T. cruzi*-infected peritoneal macrophages by plate adherence. We assessed cytotoxic activity against target macrophages using various methods as described in **Supplementary Figure 2**. **Figure 2A** shows a representative image of adherent macrophages infected with the parasites. After the co-cultures, we assessed the cytotoxicity of effector cells by: 1) evaluating the percentage of dead macrophages using aqua zombie dye, 2) counting the total number of infected target cells in the wells, and 3) counting the number of trypomastigotes (Tps) in the supernatant of the co-cultures (**Figures 2B-D**, respectively). In all cases, only T_MEM_ and T_VM_ cells were able to effectively eliminate the infected target cells to a similar extent, while T_N_ cells were not. Furthermore, co-cultures stimulated the degranulation of effector cells in the presence of target cells, compared to effector cells alone. Notably, only T_MEM_ and T_VM_ cells exhibited significantly higher levels of CD107a^+^ cells compared to T_N_ cells (**Figures 2E, F**).

**Figure 3:**
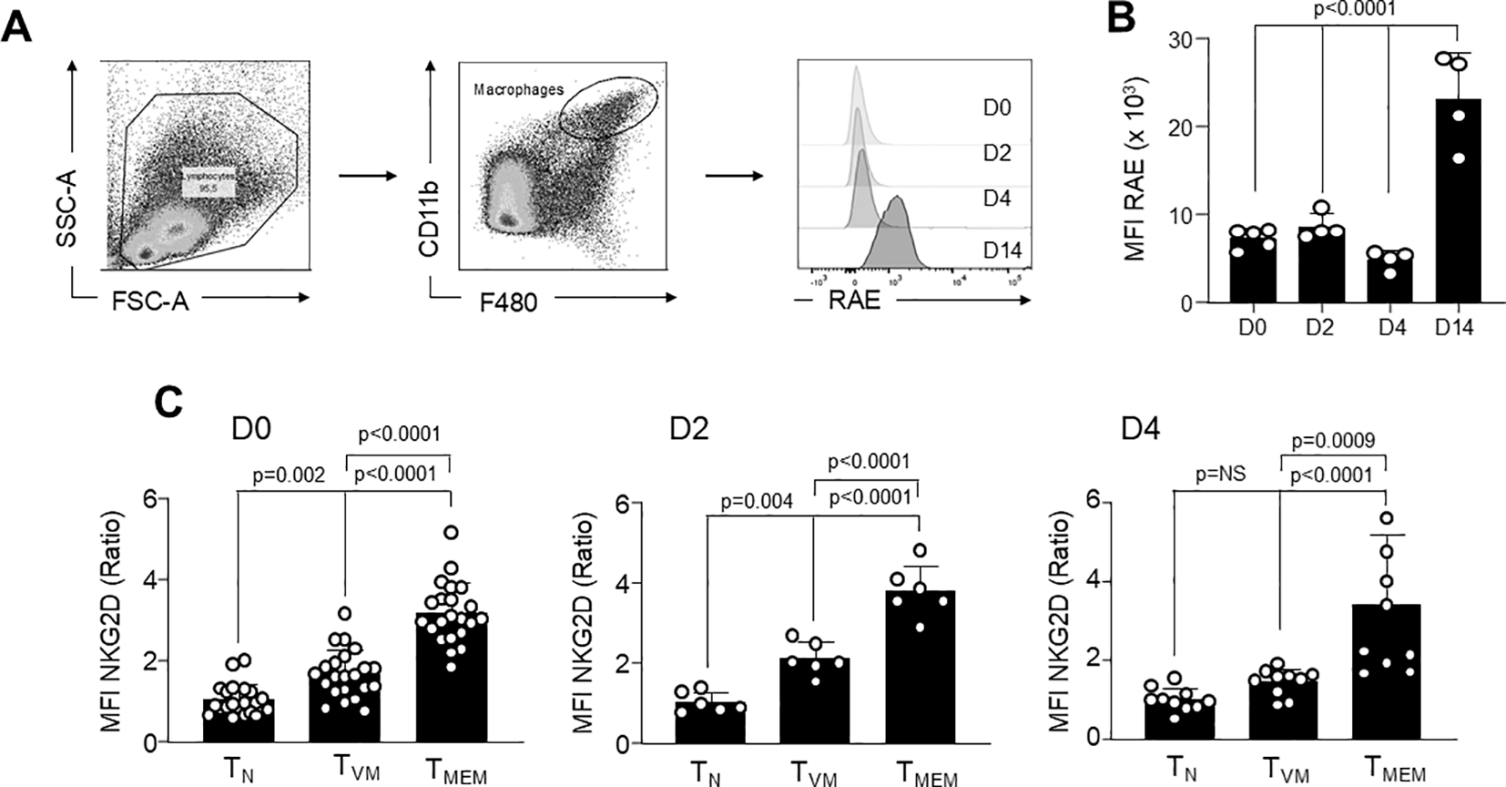
RAE expression in peritoneal macrophages and NKG2D in effector T cells remains unchanged early during *T. cruzi* infection. **(A)** Representative gating strategy for the analysis of RAE expression in PM at different days post-infection. **(B)** RAE expression (MFI) was evaluated by flow cytometry in CD11b^+^ F4/80^+^ cells at different time points post-infection. **(C)** NKG2D expression was analyzed by flow cytometry in effector T cell subsets (defined in **Figure 1**) and expressed as the ratio compared to the expression of the mean of T_N_ cells. Data are representative of 2–4 independent experiments with 3–5 mice per group. Statistical analysis was performed using one-way ANOVA. Bar graph data are shown as mean ± SEM.

One of the mechanisms operating during bystander activation of T cells is killing through the NKG2D receptor (7, 26, 27). We first evaluated the expression of one of the most common NKG2D ligands present in target cells, which is RAE (7, 26, 27), and also the expression of NKG2D on effector cells (**Figure 3**). **Figure 3A** shows the gating strategy to evaluate RAE expression on peritoneal macrophages at the same time points post-infection as in **Figure 1**. We observed that RAE expression is not upregulated early after infection, when bystander T cell activation takes place, but it is highly expressed on macrophages by day 14 post-infection (**Figures 3A, B**). Although these results do not offer a compelling explanation for cytotoxicity via this mechanism during the early days post-infection, we observed that the RAE ligand, NKG2D, is constitutively and highly expressed on T_VM_ and T_MEM_ cells compared to T_N_ cells across all time points, except for D4, where the difference between T_N_ and T_VM_ cells is not statistically significant. This finding is particularly intriguing, as T_VM_ cells levels remain stable across all time points, with no significant changes observed. In contrast, T_MEM_ cells consistently exhibits significantly higher NKG2D expression than T_VM_ cells (**Figure 3C**). Furthermore, when comparing NKG2D levels within each cell type across different time points (D0 *vs* D2 *vs* D4), no significant differences were detected. Thus, infection does not appear to further upregulate NKG2D expression during the early post-infection period (**Figure 3C**).

Despite the initial unpromising findings, we further investigated the role of NKG2D during the early stages post-*T. cruzi* infection, focusing on the bystander activation of T_MEM_ and T_VM_ cells. We evaluated the same parameters as in **Figure 2**. By using a blocking anti-NKG2D antibody in the co-cultures, we observed similar results in the presence and absence of the antibody, demonstrating that NKG2D appears to be minimally involved in controlling the parasites by these T cell types early after infection (**Supplementary Figure 3**).

**Figure 4:**
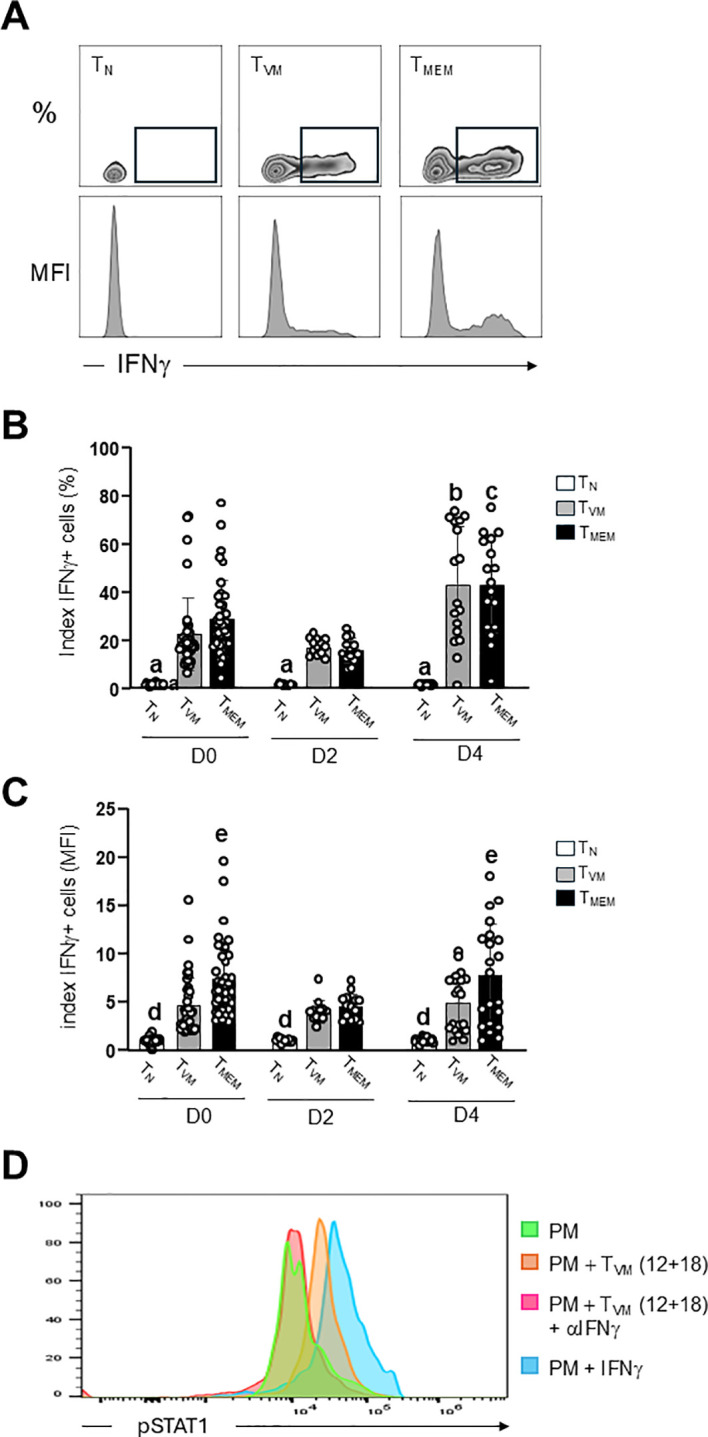
T_VM_ cells produce IFNγ and activate STAT1 signaling in *T. cruzi-*infected macrophages. **(A)** Representative dotplots (frequency) and histograms (MFI) of intracellular IFNγ in CD8^+^ T cell subsets evaluated by flow cytometry in control (non-infected, D0) and *T. cruzi*-infected mice (D2 and D4) after overnight stimulation with IL-12 and IL-18. **(B)** Frequency (%) of IFNγ^+^ cells within each subset was expressed as the index of each individual value compared to the mean of expression on T_N_ cells from control mice. Data are representative of 4 independent experiments with 3–5 mice per group. a, T_N_*vs* T_VM_ and T_MEM_ all time point, p<0.0001; b, T_VM_ D0 and D2 *vs* D4 p=0.0003 and c, T_MEM_ D0 and D2 *vs* D4 p=0.008. **(C)** MFI of IFNγ^+^ cells within each subset was expressed as the index of each individual value compared to the mean of expression on T_N_ cells from control mice. Data are representative of 4 independent experiments with 3–5 mice per group. d, T_N_*vs*. T_VM_ and T_MEM_, p=0.002 throughout the study period; e, T_MEM_*vs*. T_VM_ on D0 and D4, p=0.02. **(D)** pSTAT1 expression in PM (CD11b^+^ F4/80^+^) from IFNAR KO mice was assayed by flow cytometry after co-culture for 10 minutes with: culture medium (green histogram); supernatant from T_VM_ cells pre-stimulated with IL-12 and IL-18 in the presence (pink histogram) or absence (orange histogram) of a neutralizing anti-IFNγ antibody or supernatant of culture medium + recombinant IFNγ (light blue histogram). Data are representative of 2 independent experiments. Bar graph data are shown as mean ± SEM. Statistical analysis was performed using two-way ANOVA.

The functional mechanisms of bystander activation of T cells also involve the inflammatory cytokine IFNγ, which plays a crucial role early during infection by activating cytotoxic mediators in phagocytes, particularly macrophages (24). Moreover, T_MEM_ and T_VM_ cells are known to be prolific producers of this cytokine due to their constitutive and synergistic expression of IL-12R and IL-18R (1, 21–23, 40). In our studies, we analyzed IFNγ expression by flow cytometry, evaluating both the percentage of IFNγ^+^ cells (frequency) and the intensity of IFNγ expression (MFI) (**Figure 4A**).

When analyzing the percentage of IFNγ^+^ cells (**Figure 4B**), we observed that T_N_ cells exhibit a very low frequency of IFNγ^+^ cells, occasionally falling below the detection threshold, even when obtained from *T. cruzi*-infected mice on D2 and D4. In contrast, T_VM_ and T_MEM_ cells exhibit robust IFNγ production both in uninfected mice (D0) and at days 2 and 4 following *T. cruzi* infection. When we compared the frequency of IFNγ^+^ T_VM_ versus T_MEM_ cells at each time point, we observed similar levels between both subsets. Interestingly, by D4, both T_VM_ and T_MEM_ cells present higher frequency of cells producing IFNγ than the days before.

When evaluating the mean fluorescence intensity (MFI) of IFNγ production across different cell subsets (**Figure 4C**), we again observed that T_N_ cells exhibit consistently low levels of IFNγ expression. Interestingly, T_MEM_ cells produced significantly higher levels of IFNγ compared to T_VM_ cells on D0 and D4, although this difference was not observed on D2.

These findings indicate that both memory subsets display a high frequency of IFNγ^+^ cells; however, the intensity of IFNγ production (MFI) is generally greater in T_MEM_ cells than in T_VM_ cells, except on D2. Despite these differences in MFI, we conclude that both memory populations are robust producers of IFNγ during the early stages of *T. cruzi* infection and should be considered equally relevant as bystander sources of this cytokine.

To evaluate whether this effect extends to other infectious models, we performed experiments using intraperitoneal challenge with *Candida albicans* and assessed IFNγ expression on D2 post-infection (**Supplementary Figures 4A, B**). The results show that both T_VM_ and pre-existing T_MEM_ cells exhibit comparable capacities to produce high levels of IFNγ at early stages post-*C. albicans* infection.

To evaluate whether T_MEM_ cells’ specificity influences this phenomenon (considering that in both *T. cruzi* and *C. albicans* infections, pre-existing T_MEM_ cells represent a polyclonal population), we performed *T. cruzi* infection in OT-I mice, which are specific for the OVA antigen, not present in the parasite. Using a gating strategy similar to that shown in **Supplementary Figure 1**, we gated on OVA-specific T_N_, T_VM_, and T_MEM_ cells (**Supplementary Figure 4C**). In this model, we observed that T_VM_ and T_MEM_ cells produced elevated levels of IFNγ at day 4 post-infection, in contrast to the nearly undetectable expression in T_N_ cells (**Supplementary Figure 4D**).

These findings confirm that at early post-infection time points, both T_VM_ and T_MEM_ cells (regardless of their specificity to the pathogen) can produce high and comparable levels of IFNγ in a bystander manner, driven by stimulation with IL-12 and IL-18.

To determine whether IFNγ produced by T_VM_ cells can activate peritoneal macrophages (PM), we assessed pSTAT1 expression in target macrophages (42). Given that type I interferons also signal through STAT1 (42), we used peritoneal macrophages from IFNAR knockout (IFNAR KO) mice to exclude type I IFN-mediated effects (**Figure 4D**).

Macrophages were cultured under four different conditions, in the presence of: (1) culture medium (negative control); (2) supernatant from T_VM_ cells pre-activated with IL-12 and IL-18; (3) supernatant from IL-12/IL-18–stimulated T_VM_ cells in the presence of a neutralizing anti-IFNγ antibody and (4) culture medium + rIFNγ, serving as a positive control (**Figure 4D**).

We observed a marked increase in pSTAT1 expression following the culture supernatant from IL-12/IL-18–stimulated T_VM_ cells. However, this signal was abrogated in the presence of a neutralizing IFNγ antibody, confirming that the activation of macrophages was specifically mediated by IFNγ produced by T_VM_ cells (**Figure 4D**).

**Figure 5:**
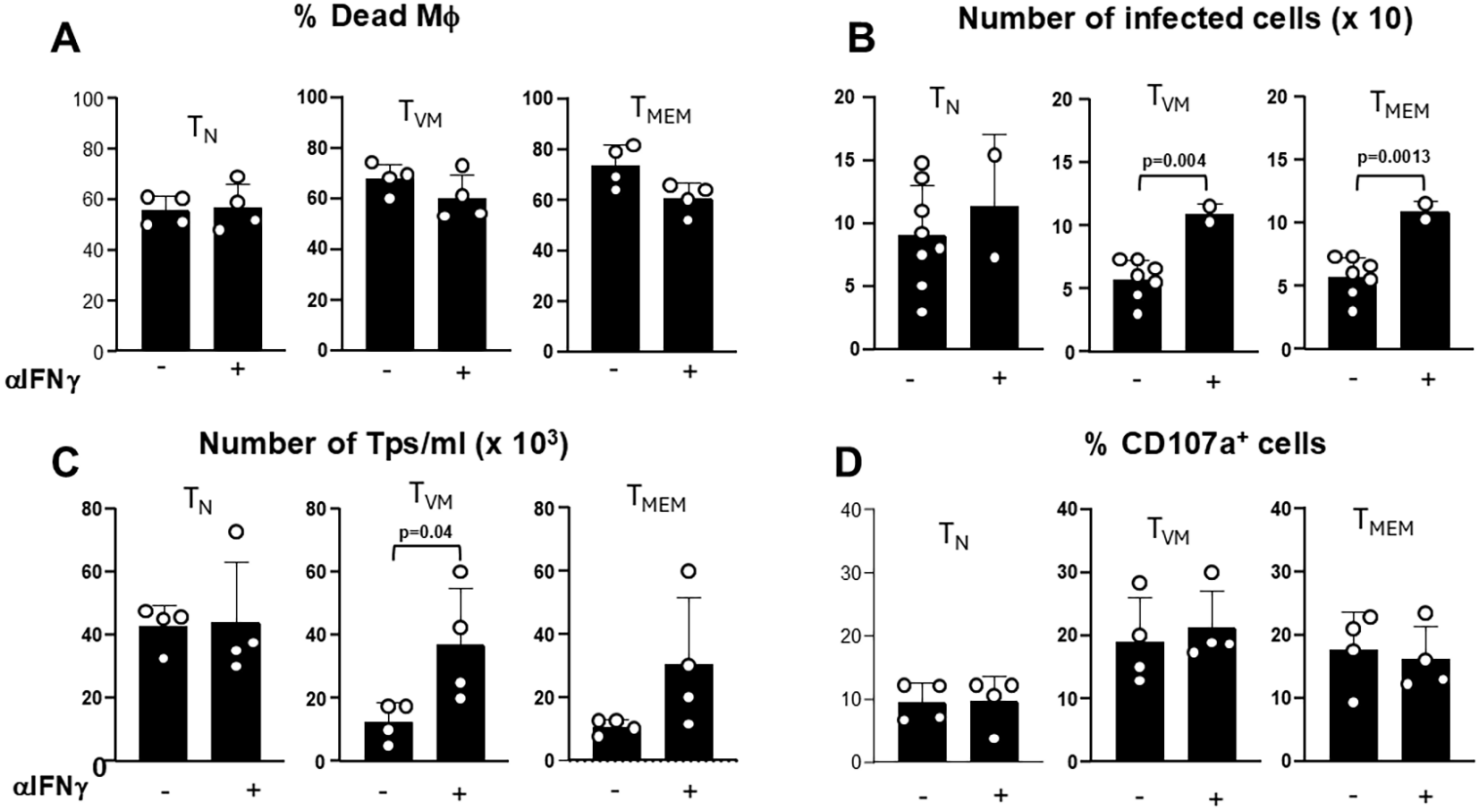
IFNγ neutralization impairs parasite control but not T cell degranulation. Cytotoxic assays were performed using sorted T_N_, T_VM_ and T_MEM_ CD8^+^ T cells pre-stimulated with IL-12 and IL-18 (effector) with enriched Tc-PM, in the presence or absence of a neutralizing anti-IFNγ antibody. **(A)** Bar graph shows the percentage of dead macrophages evaluated by aqua zombie dye in the F4/80^+^ CD11b^+^ population by flow cytometry. **(B)** Number of intracellular infected target cells, evaluated by immunofluorescence staining 48 h post-co-culture. **(C)** Number of parasites in the culture supernatants (Tps), measured 72 h after co-culture. **(D)** Flow cytometry analysis of CD107a expression in effector cells after 48 h of co-culture. Data are representative of 2 independent experiments. Statistical analysis was conducted using Student’s unpaired t-test to compare co-cultures with and without the neutralizing antibody across all cell subsets. Bar graph data are shown as mean ± SEM. When no statistically significant differences were found between groups, the corresponding information was intentionally omitted from the plot to enhance the visual clarity and highlight the biologically meaningful effects depicted in the figure.

Following these results, we performed similar cytotoxicity analyses of T_MEM_ and T_VM_ cells in the presence or absence of a neutralizing IFNγ antibody. Even though the number of dead Tc-PM remain similar with or without the neutralizing antibody (**Figure 5A**), we observed that the antibody was able to block the antiparasitic capacity observed in **Figure 2**, as evidenced by increase in the number of infected cells (**Figure 5B**) and in the number of trypomastigotes (Tps) in the supernatant in co-cultures with either T_MEM_ or T_VM_ cells (**Figure 5C**). However, the neutralization of IFNγ did not impact the degranulation capacity of these effector cells, as measured by CD107a expression after the co-cultures (**Figure 5D**).

**Figure 6:**
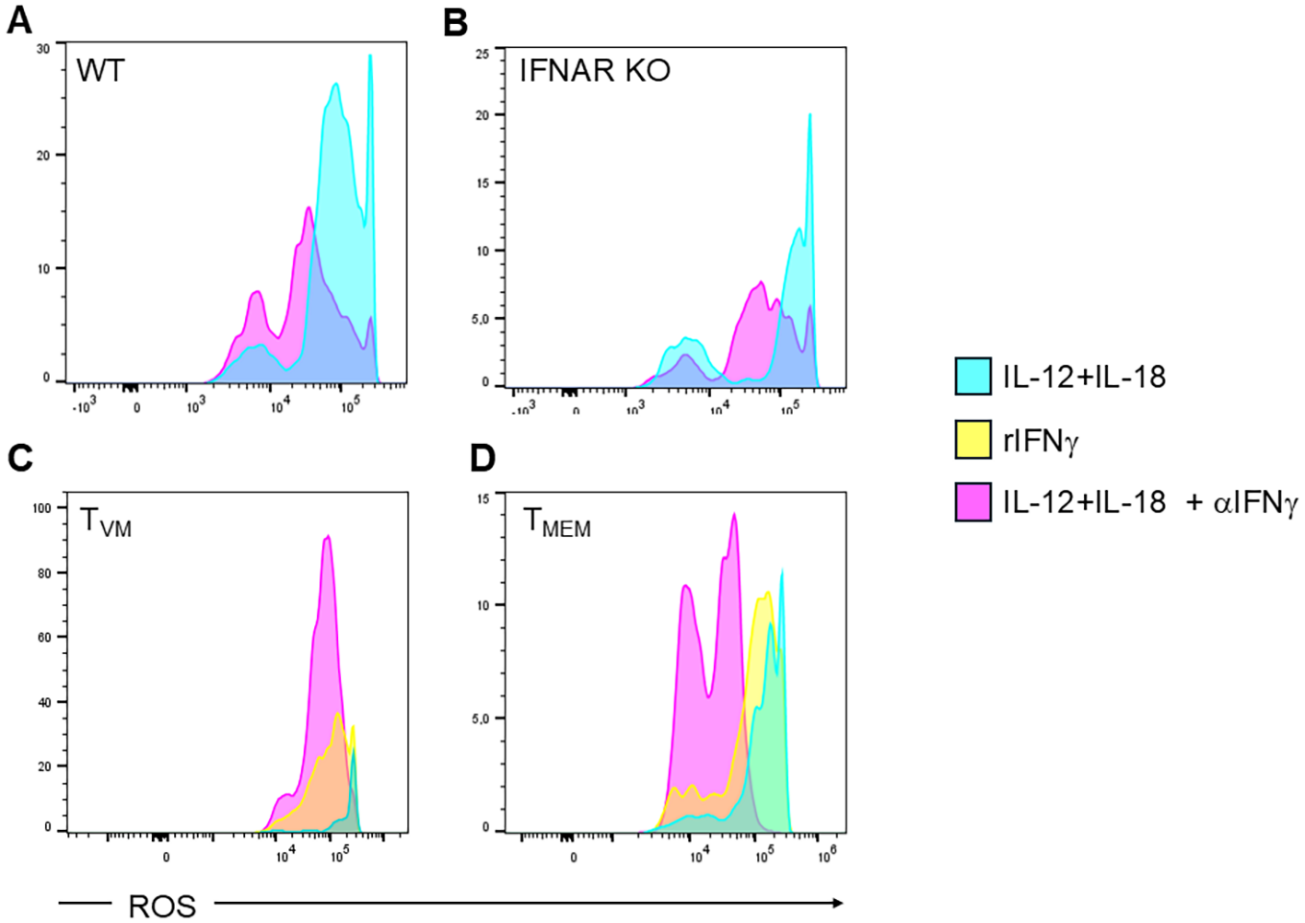
IFNγ produced by T_VM_ cells and T_MEM_ cells drives the production of reactive oxygen species (ROS) in *T. cruzi-*infected macrophages. The production of ROS in Tc-PM was evaluated by flow cytometry after 48 h of culture with the supernatant from **(A)** Splenocyte from C57BL/6 WT mice; **(B)** Splenocyte from IFNAR KO mice; sorted **(C)** T_VM_ or **(D)** T_MEM_ cells from WT all of them pre-incubated with IL-12+IL-18 in the present or absence of a neutralizing anti-IFNγ antibody. **(C, D)** As positive control, PM cells were incubated with rIFNγ. Data are representative histograms of 2 independent experiments.

**Figure 7:**
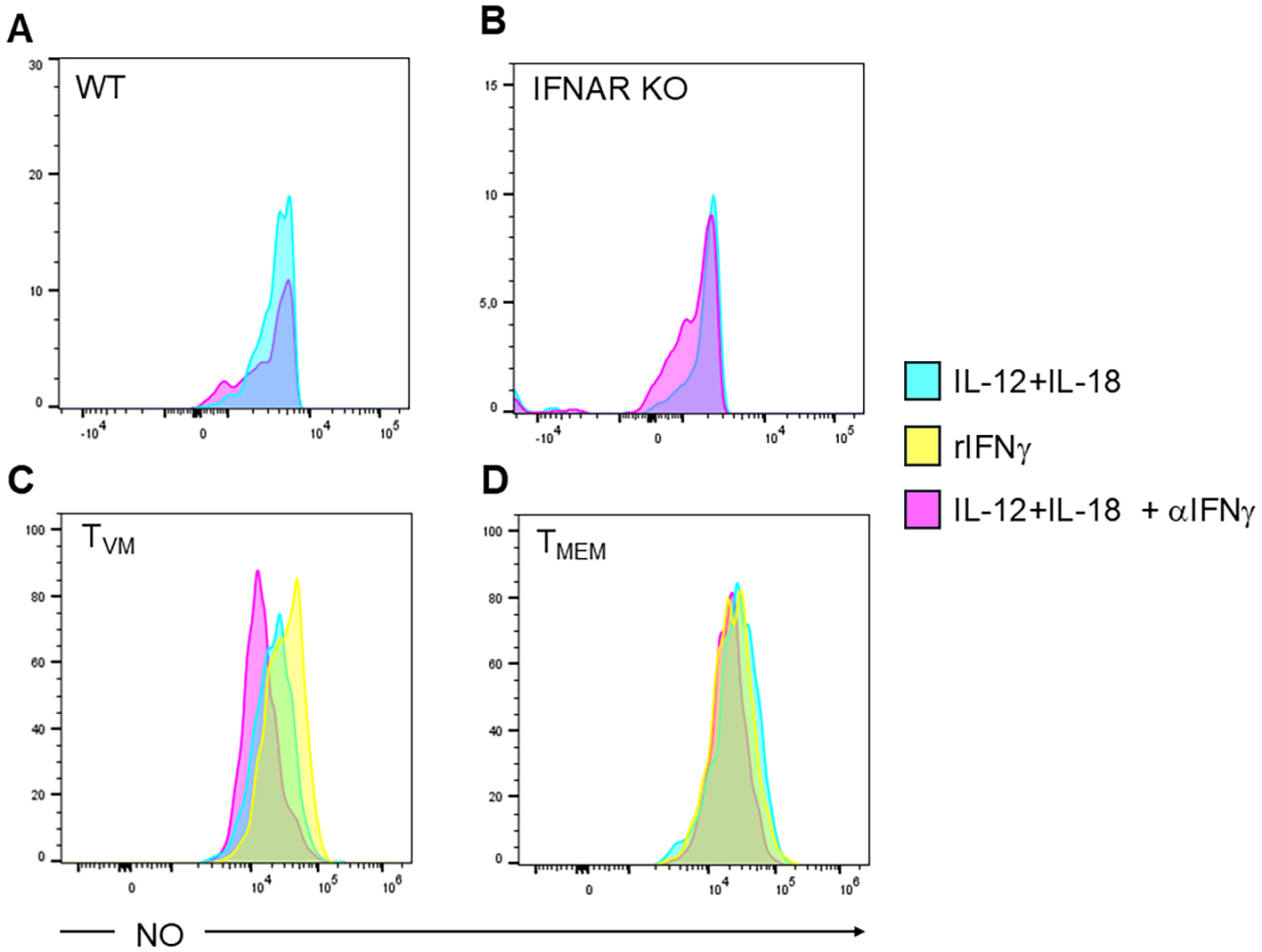
IFNγ produced by T_VM_ cells and T_MEM_ cells promote the production of nitric oxide (NO) in *T. cruzi-*infected macrophages. The production of NO in Tc-PM was evaluated by flow cytometry after 72 h of culture with the supernatant from **(A)** Splenocyte from C57BL/6 WT mice; **(B)** Splenocyte from IFNAR KO mice; sorted **(C)** T_VM_ or **(D)** T_MEM_ cells from WT all of them pre-incubated with IL-12+IL-18 in the present or absence of a neutralizing anti-IFNγ antibody. **(C, D)** As positive control, PM cells were incubated with rIFNγ. Data are representative histograms of 2 independent experiments.

Having demonstrated that T_MEM_ and T_VM_ cells produce large and similar amounts of IFNγ and that this cytokine signals in infected macrophages, we next evaluated whether the IFNγ produced by these cells through bystander activation could trigger microbicidal mechanisms such as ROS (**Figure 6**) and NO (**Figure 7**) production in target macrophages. Since type I IFNs can also activate macrophages and signal through the STAT1 molecule (42), we evaluated ROS and NO expression in target macrophages co-cultured with splenocytes from WT (**Figures 6A** and **7A**, respectively) or IFNAR KO mice (**Figures 6B** and **7B**, respectively). We observed that in both cases, these cytotoxic mechanisms are activated in a similar manner, regardless of type I IFN signaling, highlighting the major relevance of IFNγ signaling. Interestingly, the supernatants from IL-12+IL-18-stimulated T_VM_ and T_MEM_ cells strongly enhance ROS (**Figures 6C, D**, respectively) and NO (**Figures 7C, D**, respectively) production. This effect is reversed in the presence of a neutralizing IFNγ antibody, except in the case of T_MEM_ cells regarding NO production (**Figure 7D**). This suggests that T_MEM_ cells may stimulate NO through alternative signaling pathways beyond IFNγ.

**Figure 8:**
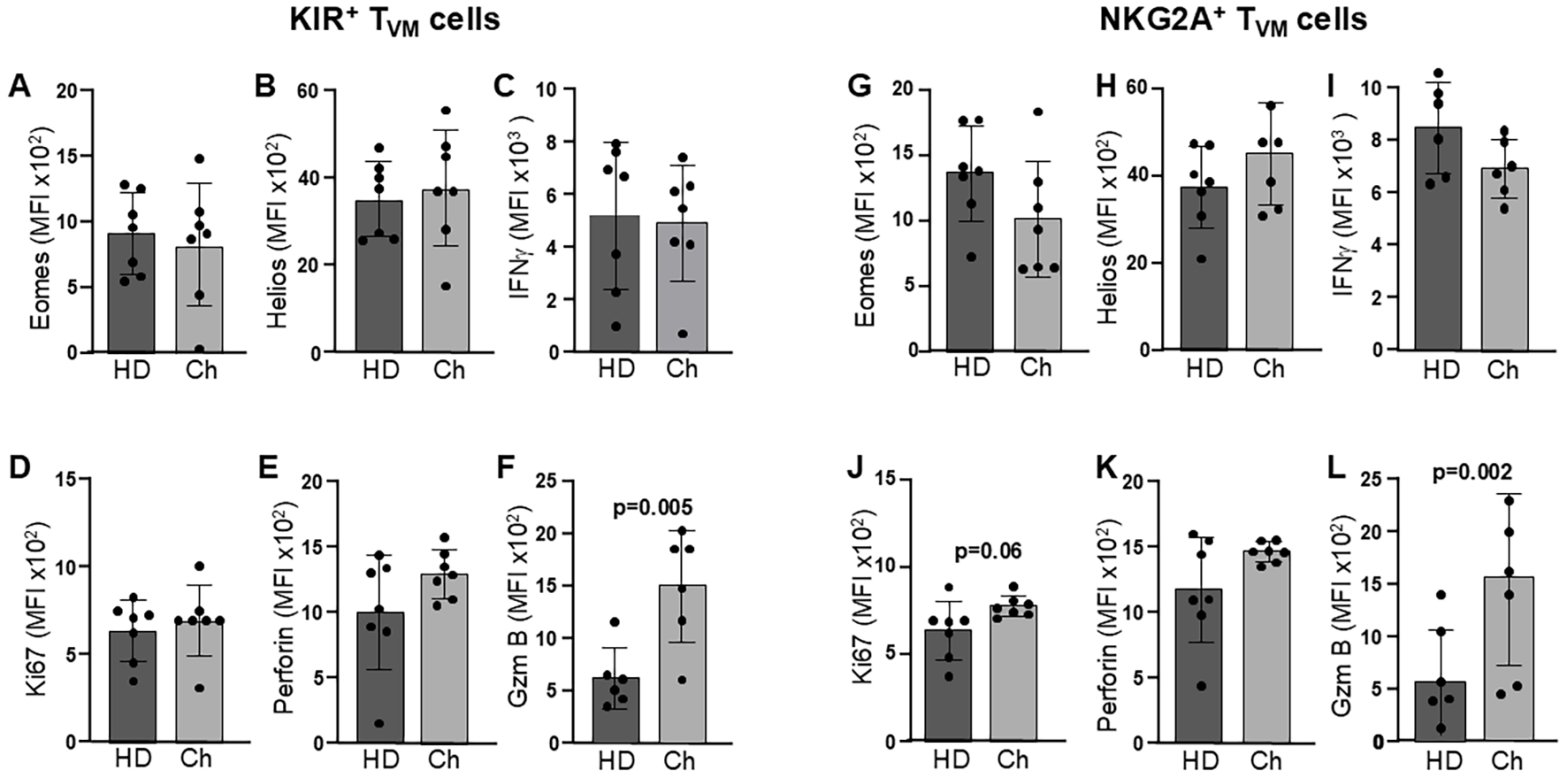
Enhanced functional profile of KIR^+^ and NKG2A^+^ human T_VM_ cells in chronic *T. cruzi* infected patients. Flow cytometry was used to evaluate the phenotypic and functional profile of KIR^+^**(A-F)** and NKG2A^+^**(G-L)** human T_VM_ cells in peripheral blood from healthy donors (HD) and Chagas patients (Ch). Shown are the Mean Fluorescence Intensity (MFI) of PMA/ionomycin-stimulated cells expressing the transcription factors Eomes **(A, G)** and Helios **(B, H)**, the cytokine IFNγ **(C, I)**, Ki67 as a proliferation marker **(D, J)**, Perforin **(E, K)** and Granzyme B **(F, L)** as cytotoxic effector markers. A total of 7 HD and 7 Chagas patients were evaluated for statistical purposes. Statistical analysis was conducted using Student’s unpaired t-test. Bar graph data are shown as mean ± SD.

Virtual memory T cells also exist in humans and share many similarities with their murine counterparts (32, 43, 44). Moreover, bystander activation can also occur in chronic infections in both mice and humans (3, 45). Unfortunately, it is very difficult to identify Chagas patients in the acute phase of *T. cruzi* infection, as most patients only become aware of their condition years after the infection. Despite these challenges, we found it very interesting to evaluate the functional state of human T_VM_ cells in chronically Chagas patients, as this has not been reported thus far. Two types of human T_VM_ cells with different functional properties have been reported: KIR^+^ T_VM_ and NKG2A^+^ T_VM_ (32). We have analyzed several functional parameters in both types of cells in chronically *T. cruzi*-infected patients (**Figures 8**). To analyze human T_VM_ cells, we employed an elimination gating strategy to exclude other innate cell types such as NK, NKT, γδ T cells and MAIT CD8^+^ T cells, as shown in **Supplementary Figure 5A** and similarly as reported by other investigators (32). We first analyzed the frequency of KIR^+^ and NKG2A^+^ cells in healthy donors (HD) and Chagas patients (Ch). While the incidence of NKG2A^+^ cells was comparable between groups, the frequency of KIR^+^ cells was higher in Chagas patients, showing a trend toward statistical significance. (**Supplementary Figure 5B**). We found that NKG2A^+^ T_VM_ cells from Chagas patients showed a trend toward higher Ki67 expression compared to HDs, with the difference approaching statistical significance. However, this difference is relatively small and may or may not carry biological significance (**Figure 8**). On the other hand, when examining functional markers, we observed that perforin levels were marginally elevated in Chagas patients, although the difference was not statistically significant. In contrast, granzyme B levels were significantly higher in *T. cruzi*-infected individuals (**Figure 8**). Surprisingly, no significant differences were observed in IFNγ levels between healthy donors and Chagas patients nor in the expression levels of the transcription factors (TFs) Eomes and Helios (**Figure 8**).

**Figure 9:**
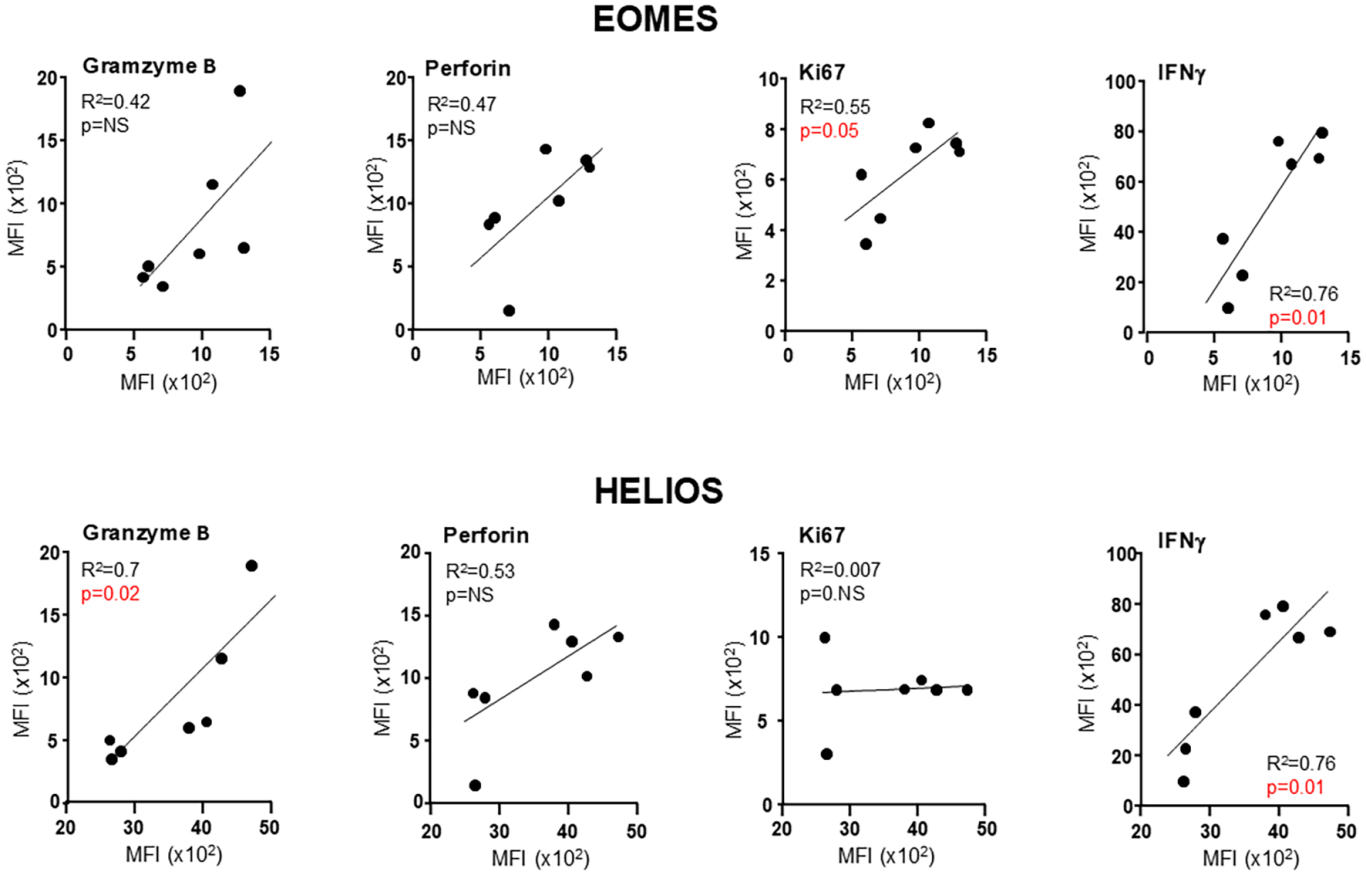
Functional markers in KIR^+^ T_VM_ cells from healthy donors correlate with the expression of Eomes and Helios. Representative simple linear regression analyses illustrating the association between the mean fluorescence intensity (MFI) of Eomes or Helios with MFI of functional markers (granzyme B, perforin, Ki67, and IFNγ) in KIR^+^ T_VM_ cells from healthy donors. Each dot represents an individual donor. Coefficients of determination (R²) and p-values were calculated using simple linear regression analysis.

Given the high variability typically observed in studies involving human samples, combined with the limited size of our cohort, it is likely that certain biologically relevant effects may be obscured when analyzing the direct expression of individual parameters. To further explore the data obtained from human T_VM_ cells, we performed linear regression analyses (LRA) between various functional markers (granzyme B, perforin, Ki67, and IFNγ) versus the reported transcription factor Eomes, which is expressed in human T_VM_ cells (43, 44). We also assessed the transcription factor Helios, previously described in CD8^+^ KIR^+^ cells with a regulatory function, although its expression in KIR^+^ human T_VM_ cells remains unexplored (46, 47). As an illustrative example, we present the LRA of KIR^+^ T_VM_ cells from healthy individuals (**Figure 9**), while the complete analysis can be found in **Supplementary Figure 6**.

Breaking down the most striking findings, we observed a positive correlation between Ki67 levels and Eomes expression in KIR^+^ cells (**Supplementary Figure 6A**), as well as with Helios expression in NKG2A^+^ cells from healthy donors (HDs) (**Supplementary Figure 6B**). Notably, these associations were absent in *T. cruzi*-infected patients. Regarding perforin expression, we found strong correlations with both Eomes and Helios in KIR^+^ cells from HDs and Chagas patients (**Supplementary Figure 6A**), but no such correlation was detected in NKG2A^+^ cells (**Supplementary Figure 6B**). As for granzyme levels, a significant correlation with both transcription factors was observed in KIR^+^ cells from HDs (**Supplementary Figure 6A**), while in NKG2D^+^ cells, only Helios showed a significant association (**Supplementary Figure 6B**). Importantly, given the markedly elevated granzyme levels in *T. cruzi*-infected individuals across both KIR^+^ and NKG2A^+^ subsets (**Figures 8F, L**), these correlations were even more pronounced in Chagas patients (**Supplementary Figures 6A, B**). Unexpectedly, no significant differences in IFNγ levels were observed between HDs and Chagas patients within either the KIR^+^ or NKG2A^+^ cell subsets. However, we found a strong correlation between IFNγ expression and both transcription factors across the two cell types, suggesting that this functional parameter is closely linked to the core transcriptional profile of human T_VM_ cells. In the case of human samples, we chose to employ a broader stimulation approach using PMA/Ionomycin rather than cytokine-based stimulation, given the differential expression of cytokine receptors between KIR^+^ and NKG2A^+^ cells (32), which could selectively activate one subset over the other. Under this stimulation, we consistently observed that NKG2A^+^ cells were more prone to express higher levels of IFNγ than KIR^+^ cells in both HDs and Chagas patients (**Supplementary Figure 6C**).

These findings demonstrate, for the first time, that this subset of CD8^+^ T cells display an enhanced functional phenotype (marked by elevated granzyme expression) in chronically infected Chagas patients compared to healthy controls. Furthermore, these functional parameters may differentially correlate with the expression levels of the key transcription factors Eomes and Helios in KIR^+^ versus NKG2A^+^ T_VM_ cells.

## Discussion

Bystander activation of T cells refers to the phenomenon in which T cells become activated without direct recognition of their specific antigen through the TCR. Instead, these cells respond to inflammatory signals, such as cytokines like IL-12 and IL-18, during infections or immune responses. This activation allows T cells to rapidly contribute to host defense by producing cytokines like IFNγ, enhancing the immune response, and supporting the activation of other immune cells, including macrophages and dendritic cells (3, 4, 16, 48). This mechanism is particularly relevant in infections caused by intracellular pathogens, such as *Trypanosoma cruzi*, where broad immune activation is necessary to control microbial replication and spread (2).

In this context, extensive research has explored the role of pre-formed T_MEM_ cells in the bystander phenomenon (3). While T_VM_ cells are capable of mediating robust immunological protection against pathogens even in the absence of their cognate antigen, the functional advantages associated with their high prevalence in the immune repertoire remain to be fully elucidated within the bystander activation mechanism.

The ability of T_VM_ cells to provide bystander protection was documented upon the transfer of monoclonal gBT-1 T_VM_ cells into irrelevant MHC I-restricted TCR transgenic mice and subsequent infection with *Listeria monocytogenes (*23). Aside from this report, only a few additional papers demonstrate bystander protection of T_VM_ cells *in vivo* against infectious pathogens, including our own study (10, 31, 49). Moreover, only 2 studies have evaluated the differences between the gene expression programs of T_MEM_ versus T_VM_ cells, which might influence the efficiency of their bystander protection on an *in vivo* setting (15, 16). These studies demonstrate that the transcriptional signature of T_VM_ cells is highly similar to that of T_MEM_ cells, expressing a wide array of functional genes. These include NK-related genes, cell-killing genes, inflammatory cytokines and chemokines, and cytokine sensing genes. In contrast, T_N_ cells do not express any of these genes involved in the cytotoxic response of CD8^+^ T cells (15, 16). Most of these differences in gene expression have not been confirmed at the protein level, nor have their impact on the cytotoxic capacity of T_VM_ versus T_MEM_ cells been established. Only one study has evaluated the differences between T_N_, T_VM_, and T_MEM_ cells at an *in vivo* and *in vitro* setting, focusing on an age-related context, and demonstrated that aged T_VM_ cells exhibit a profile consistent with senescence (50).

Bystander activation functions primarily through the activation of cytolytic mechanisms via cytokines (mainly IL-12, IL-18, and IL-15) as well as through the NKG2D receptor in the absence of TCR stimulation in cells expressing its ligands, particularly in infected and neoplastic cells (45).

For instance, *in vivo* blockade of NKG2D-NKG2DL interactions has been shown to result in increased bacterial loads early after infection, even in the absence of NK cells. This finding suggests that bystander-activated T cells play a crucial role in eliminating *L. monocytogenes*-infected APCs expressing NKG2DLs (7). Additionally, while T_VM_ cells express NKG2D at lower levels compared to T_MEM_ cells, they do so at higher levels than T_N_ cells (16). In this context, we sought to investigate the role of NKG2D by T_MEM_ and T_VM_ cells in the early stages of *T. cruzi* infection. Our data indicates that, although NKG2D is expressed in both types of memory cells at different extend, its blockade does not appear to impact the clearance of *T. cruzi* in infected peritoneal macrophages. We speculate that this may be due to the low expression of RAE (and potentially other NKG2DLs) in *T. cruzi*-infected macrophages at early time points as visualized in **Figure 3**. This reduced expression likely renders alternative bystander mechanisms responsible for the highly cytotoxic activity observed in the effector:target co-cultures.

One of the most abundant cytokines produced by bystander T_MEM_ and T_VM_ cells is IFNγ, as both types of cells constitutively express IL-12 and IL-18 receptors, which act synergistically to induce its production (2–4, 16).

Interestingly, not only early IFNγ production by T_VM_ cells could induce protection against pathogens but recent demonstrations have shown that IFNγ produced by T_VM_ cells, but not other sources, can shape the subsequent adaptive immune response. This is achieved by promoting the expansion of low-avidity T cells while reinforcing the entry of high-avidity T cells into the memory pool. As a result, the average avidity of the primary response is reduced, and that of the memory response is increased (51).

Our work demonstrates not only that T_VM_ and pre-existing T_MEM_ cells produce high and comparable levels of IFNγ following stimulation with IL-12 and IL-18 at early post-infection time points in two distinct infectious models, but also that the antigen specificity of both T_VM_ and T_MEM_ cells is independent of this bystander activation phenomenon. The presence of T_MEM_ cells in unmanipulated OT-I mice has been recently reported. In this study, the authors used an improved method to accurately measure OT-I TCR binding affinity to 19 peptides, including foreign and self-antigens. In contrast to previous results and like other TCRs, they found that OT-I T cells display enhanced but imperfect discrimination, enabling them to be activated by high levels of self-antigen (52).

Our work demonstrates that IFNγ produced by T_VM_ cells is sufficient to activate the STAT1 signaling pathway in *T. cruzi*-infected macrophages. Moreover, IFNγ, but not type I IFNs, induces the production of ROS and NO, two of the most critical cytolytic mechanisms in macrophages.

Virtual memory T cells also exist in humans (32), yet their role in infectious processes remains largely unexplored. In this study, we provide evidence of their phenotype and functional characteristics in chronically *T. cruzi*-infected patients. Furthermore, only one report has examined the potential role of T_VM_ cells compared to virus-specific memory T cells in human infectious diseases, such as HIV infection. This study found that human T_VM_ cells, but not HIV-specific CD8^+^ T cells, may contribute to the mechanism that restricts the HIV DNA reservoir in HIV-infected individuals undergoing antiretroviral therapy (53).

Our data demonstrates that human T_VM_ cells in the blood of Chagas patients exhibit an increase in the KIR^+^ population, which did not reach statistical significance likely due to the limited cohort size in this study. Interestingly, in other infectious diseases such as influenza and SARS-CoV-2, the proportion of KIR^+^ CD8^+^ T cells increase and has even been shown to correlate with disease severity in SARS-CoV-2 infection (46). Notably, our findings reveal a more cytotoxic profile in T_VM_ cells from Chagas patients compared to healthy individuals, primarily driven by elevated granzyme expression in both KIR^+^ and NKG2A^+^ T_VM_ subsets. To further analyze human data, we performed a correlation analysis and, for the first time, identified a direct association between functional parameters and the transcription factor Eomes, previously reported to be expressed in human T_VM_ cells (43, 44). Additionally, we included, for the first time in human T_VM_ cells, the evaluation of Helios, a transcription factor previously identified in KIR^+^ CD8^+^ T cells with immunoregulatory activity in both humans and mice (46, 47, 54). In humans, *in vitro* studies have shown that KIR^+^ CD8^+^ T cells can specifically eliminate gliadin-specific pathogenic CD4^+^ T cells in celiac disease through class I MHC–dependent cytotoxicity (46). In other report, the authors demonstrate a marked upregulation of cytotoxic molecules such as granzyme B and perforin, along with the transcription factor Helios. Although the authors do not classify these cells as T_VM_ cells and do not address whether these cells co-express Eomes (55).

Our findings suggest that human T_VM_ cells, that express Helios and Eomes, show a high correlation with effector mediators as Granzyme B, perforin and IFNγ especially in Chagas patients. If these cells participate in the control of autoreactive cells is still unknown and warrants further investigation in diverse physiological and pathological contexts.

Consistent with our data, previous studies have demonstrated that a subset of human NKG2A^+^ CD8^+^ T cells capable of producing IFNγ can inhibit the *in vitro* intracellular growth of *Mycobacterium tuberculosis* in macrophages (56).

In the specific case of *T. cruzi* infection, CD8+ T cells has been associated to protection as well as the development of Chagas-related cardiomyopathy due to heart-infiltrating CD8^+^ T cells in both human and murine models (57–59). Given the strong positive correlation observed between the expression of transcription factors (TFs) and functional parameters (TFs/FP) in clinically asymptomatic Chagas patients, future clinical assessments of TFs/FP levels in T_VM_ cells may prove highly valuable for predicting either the onset of infection-associated symptoms or, conversely, the development of a more potent antiparasitic immune response.

Regarding this point, we recognize that expanding the cohort would enhance the statistical power and biological significance of the results.

We also acknowledge that certain murine experiments involved substantial technical complexity, requiring a large number of animals to obtain adequately sorted populations of T_N_, T_VM_, and T_MEM_ cells for downstream analyses. While this represents a logistical challenge in terms of scalability and reproducibility, the approach was essential to ensure the robustness and reliability of the data generated.

The data presented in this study clearly support, not discredit, the established role of T_MEM_ cells in bystander activation, particularly during the early stages of infection. Our aim, rather, is to highlight that T_VM_ cells are highly abundant in unprimed mice and can efficiently control the spread of *T. cruzi* within the first week post-infection. This control is achieved through the activation of macrophage-mediated protective immune mechanisms, with a level of effectiveness comparable to that of T_MEM_ cells.

## Data Availability

The original contributions presented in the study are included in the article/**Supplementary Material**. Further inquiries can be directed to the corresponding author.

## Supplementary material

Supplementary Figure 1 Flow cytometry gating strategy used to identify T_N_, T_VM_ and T_MEM_ CD8^+^ T cell subsets. Representative flow cytometry plots illustrating the gating strategy used to evaluate the different population of CD8^+^ cells in spleen from B6 mice. Initial gates were set on forward and side scatter (FSC/SSC) to select the lymphocyte population, followed by exclusion of doublets and dead cells in the CD8^+^ subset. Finally, we identified T_N_, T_MEM_ and T_VM_ subsets based on the expression of CD44 and CD49d.

Supplementary Figure 2 Experimental model designed to evaluate the cytotoxic activity of T_N_, T_VM_ and T_MEM_ cells against *T. cruzi*-infected macrophages. Schematic representation of the experimental setup used to evaluate the effector function of the different subsets of CD8^+^ cells. Briefly, T_N_, T_VM_, and T_MEM_ cells were sorted from C57BL/6 mice. Then, cells were stimulated overnight (ON) with IL-12 and IL-18 and co-cultured with in the presence or absence of IFNγ or NKG2D blocking antibodies. The cytotoxic response was evaluated by measuring CD107a expression (as an indicator of degranulation), counting *T. cruzi*-infected cells, quantifying extracellular *T. cruzi* parasites in the supernatants (SN) and assessing the mortality of Tc-PM.

Supplementary Figure 3 Blockade of NKG2D does not affect effector T_VM_ and T_MEM_ cell-mediated parasite control. Cytotoxic assays were performed using sorted T_N_, T_VM_ and T_MEM_ CD8^+^ T cells pre-stimulated with IL-12 and IL-18 (effector) with enriched Tc-PM, in the presence or absence of a neutralizing anti-NKG2D antibody. **(A)** Bar graph shows the percentage of dead macrophages evaluated by aqua zombie dye in the F4/80^+^ CD11b^+^ population by flow cytometry. **(B)** Number of intracellular infected target cells, evaluated by immunofluorescence staining 48 h post-co-culture. **(C)** Number of parasites in the culture supernatants (Tps), measured 72 h after co-culture. **(D)** Flow cytometry analysis of CD107a expression in effector cells after 48 h of co-culture. Data are representative of 2 independent experiments. Statistical analysis was conducted using Student’s unpaired t-test to compare co-cultures with and without the neutralizing antibody across all cell subsets. Bar graph data are shown as mean ± SEM.

Supplementary Figure 4 Early IFNγ expression by T_N_, T_VM_ and pre-existing T_MEM_ cells in different infection models. The frequency of IFNγ^+^ cells by the different CD8^+^ T cell subsets was assessed by flow cytometry in *C.albicans***(A, B)** or *T. cruzi*-OT I **(C, D)** infected mice. **(A)** Representative flow cytometry plots and **(B)** Quantification of IFNγ^+^ cell frequency within T_N_, T_VM_, and T_MEM_ subsets from *Candida albicans*-infected mice at day 2 post-infection. **(C)** Gating strategy used to identify OVA tetramer^+^ (OVAt^+^) cells in T_N_, T_VM_, and T_MEM_ populations of *T. cruzi*-infected mice on day 4 post-infection. **(D)** Frequency of IFNγ^+^ cells in OVAt^+^ T_N_, T_VM_, and T_MEM_ cells of *T. cruzi*-infected mice on day 4 post-infection. Data are representative of 2 independent experiments. Statistical analysis was performed using one-way ANOVA).

Supplementary Figure 5 Flow cytometry gating strategy and frequency of human KIR^+^ and NKG2A^+^ T_VM_ cells. **(A)** Representative flow cytometry plots illustrating the gating strategy. Initial gates were set on forward and side scatter (FSC/SSC) to select the lymphocyte population, followed by exclusion of doublets and dead cells in the CD8^+^ subset. Cells expressing markers for NKT (TCRVα24), MAIT (TCRVα7.2) were then excluded as well as TCRαβ^neg^ cells. Finally, analysis was performed on the remaining conventional CD8^+^ T cells for KIR and NKG2A expression. **(B)** Frequency of KIR^+^ T_VM_ cells and NKG2A^+^ T_VM_ cells in healthy donor (HD) and Chagas patients (Ch). Statistical analysis was performed by using Student’s unpaired t-test.

Supplementary Figure 6 Correlation of transcription factors and effector molecules expression in NKG2A^+^ and KIR^+^ T_VM_ CD8^+^ T cells from healthy and *T. cruzi*-infected individuals. Correlations between the expression of the transcription factors Eomes or Helios and cytotoxic/activation markers (Granzyme B, Perforin, Ki67, IFNγ). R² and p-values were determined using simple linear regression analysis in **(A)** KIR^+^ and **(B)** NKG2A^+^ human T_VM_ cells from healthy donors (HD) and Chagas patients (Ch patients). **(C)** Mean fluorescence intensity (MFI) of IFNγ^+^ cells in KIR^+^ and NKG2A^+^ cells between HD and Ch patients. Statistical significance was assessed by two-way ANOVA.
