# Supplementary figures and images for: Bystander CD8+ conventional memory versus virtual memory T cells in the initial days post-Trypanosoma cruzi infection
Source: Front Immunol. 2025 Dec 8;16:1674964. doi: 10.3389/fimmu.2025.1674964 (PMC12719477; doi:10.3389/fimmu.2025.1674964)

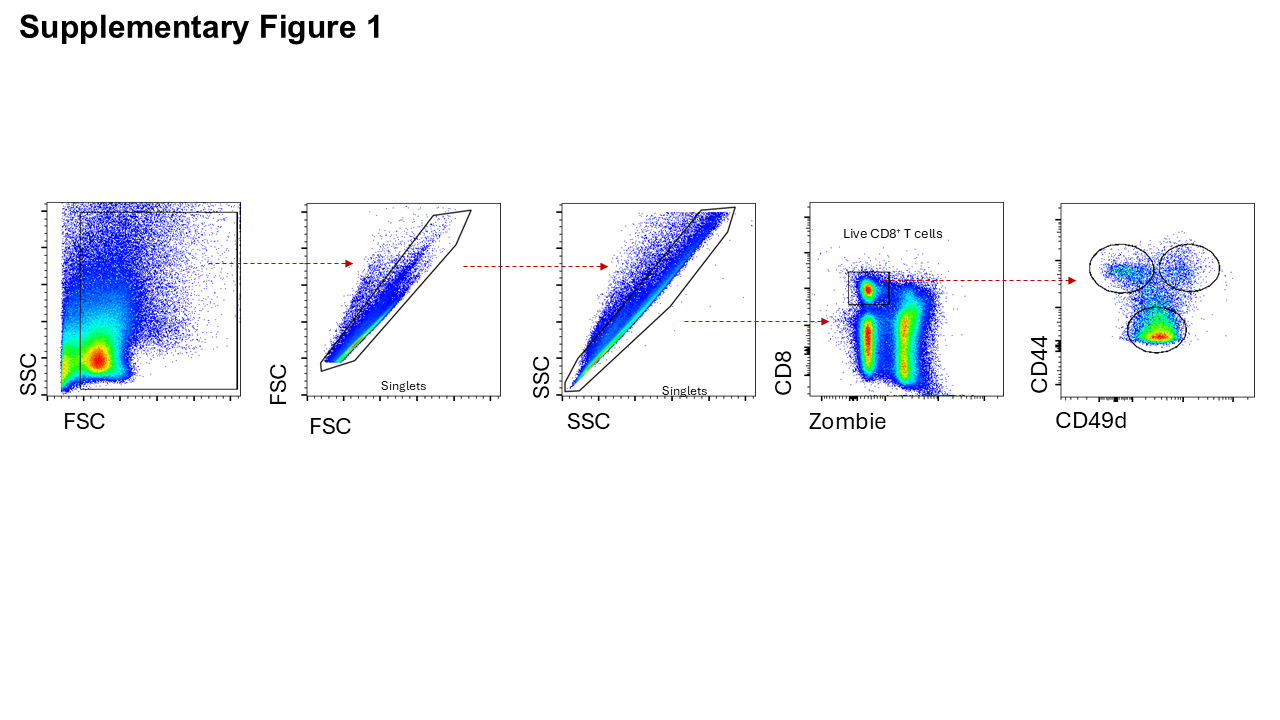

Supplement: Supplementary Figure 1 — Flow cytometry gating strategy used to identify TN, TVM and TMEM CD8+ T cell subsets. Representative flow cytometry plots illustrating the gating strategy used to evaluate the different population of CD8+ cells in spleen from B6 mice. Initial gates were set on forward and side scatter (FSC/SSC) to select the lymphocyte population, followed by exclusion of doublets and dead cells in the CD8+ subset. Finally, we identified TN, TMEM and TVM subsets based on the expression of CD44 and CD49d. [file Image1.tif]

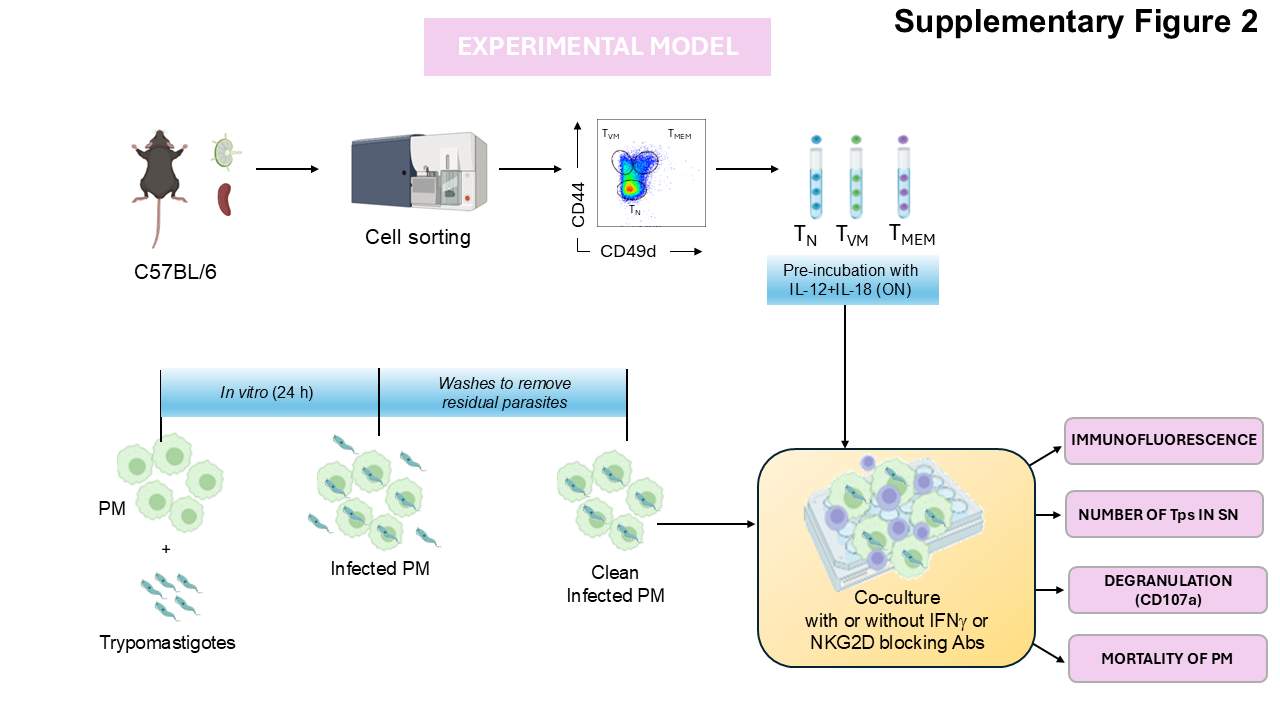

Supplement: Supplementary Figure 2 — Experimental model designed to evaluate the cytotoxic activity of TN, TVM and TMEM cells against T. cruzi-infected macrophages. Schematic representation of the experimental setup used to evaluate the effector function of the different subsets of CD8+ cells. Briefly, TN, TVM, and TMEM cells were sorted from C57BL/6 mice. Then, cells were stimulated overnight (ON) with IL-12 and IL-18 and co-cultured with in the presence or absence of IFNγ or NKG2D blocking antibodies. The cytotoxic response was evaluated by measuring CD107a expression (as an indicator of degranulation), counting T. cruzi-infected cells, quantifying extracellular T. cruzi parasites in the supernatants (SN) and assessing the mortality of Tc-PM. [file Image2.tif]

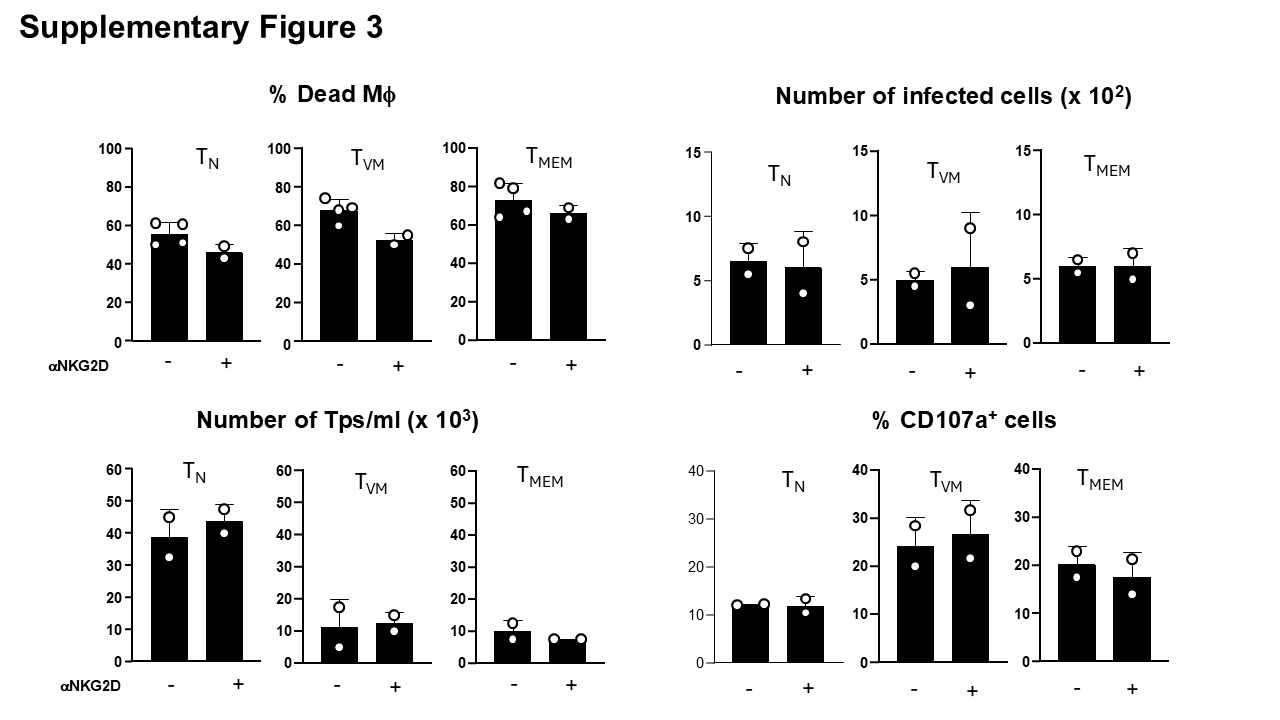

Supplement: Supplementary Figure 3 — Blockade of NKG2D does not affect effector TVM and TMEM cell-mediated parasite control. Cytotoxic assays were performed using sorted TN, TVM and TMEM CD8+ T cells pre-stimulated with IL-12 and IL-18 (effector) with enriched Tc-PM, in the presence or absence of a neutralizing anti-NKG2D antibody. (A) Bar graph shows the percentage of dead macrophages evaluated by aqua zombie dye in the F4/80+ CD11b+ population by flow cytometry. (B) Number of intracellular infected target cells, evaluated by immunofluorescence staining 48 h post-co-culture. (C) Number of parasites in the culture supernatants (Tps), measured 72 h after co-culture. (D) Flow cytometry analysis of CD107a expression in effector cells after 48 h of co-culture. Data are representative of 2 independent experiments. Statistical analysis was conducted using Student’s unpaired t-test to compare co-cultures with and without the neutralizing antibody across all cell subsets. Bar graph data are shown as mean ± SEM. [file Image3.tif]

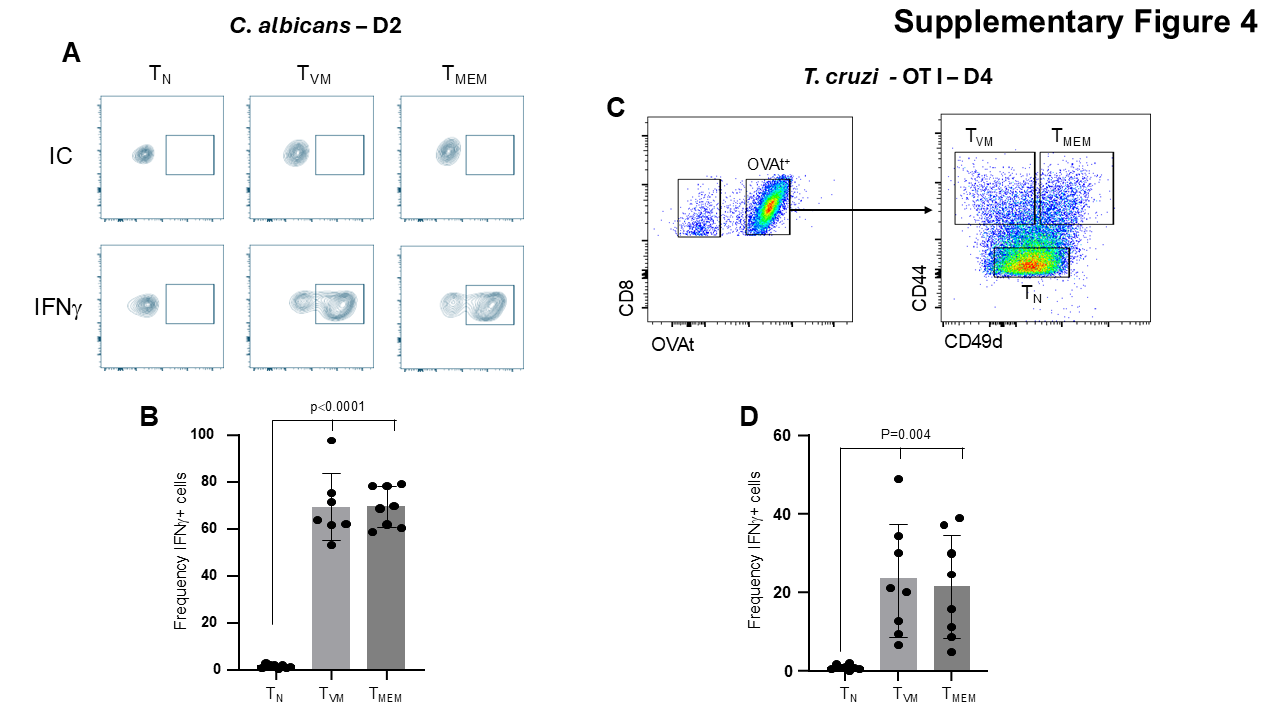

Supplement: Supplementary Figure 4 — Early IFNγ expression by TN, TVM and pre-existing TMEM cells in different infection models. The frequency of IFNγ+ cells by the different CD8+ T cell subsets was assessed by flow cytometry in C.albicans(A, B) or T. cruzi-OT I (C, D) infected mice. (A) Representative flow cytometry plots and (B) Quantification of IFNγ+ cell frequency within TN, TVM, and TMEM subsets from Candida albicans-infected mice at day 2 post-infection. (C) Gating strategy used to identify OVA tetramer+ (OVAt+) cells in TN, TVM, and TMEM populations of T. cruzi-infected mice on day 4 post-infection. (D) Frequency of IFNγ+ cells in OVAt+ TN, TVM, and TMEM cells of T. cruzi-infected mice on day 4 post-infection. Data are representative of 2 independent experiments. Statistical analysis was performed using one-way ANOVA). [file Image4.tif]

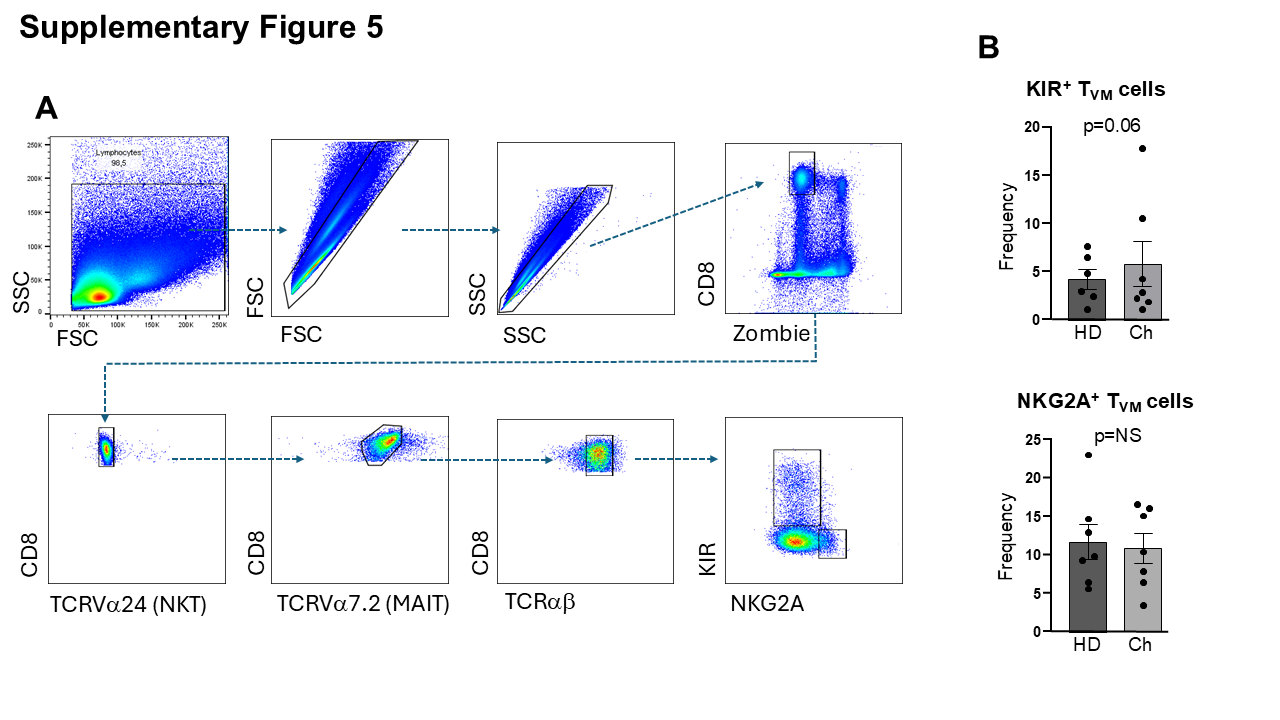

Supplement: Supplementary Figure 5 — Flow cytometry gating strategy and frequency of human KIR+ and NKG2A+ TVM cells. (A) Representative flow cytometry plots illustrating the gating strategy. Initial gates were set on forward and side scatter (FSC/SSC) to select the lymphocyte population, followed by exclusion of doublets and dead cells in the CD8+ subset. Cells expressing markers for NKT (TCRVα24), MAIT (TCRVα7.2) were then excluded as well as TCRαβneg cells. Finally, analysis was performed on the remaining conventional CD8+ T cells for KIR and NKG2A expression. (B) Frequency of KIR+ TVM cells and NKG2A+ TVM cells in healthy donor (HD) and Chagas patients (Ch). Statistical analysis was performed by using Student’s unpaired t-test. [file Image5.tif]

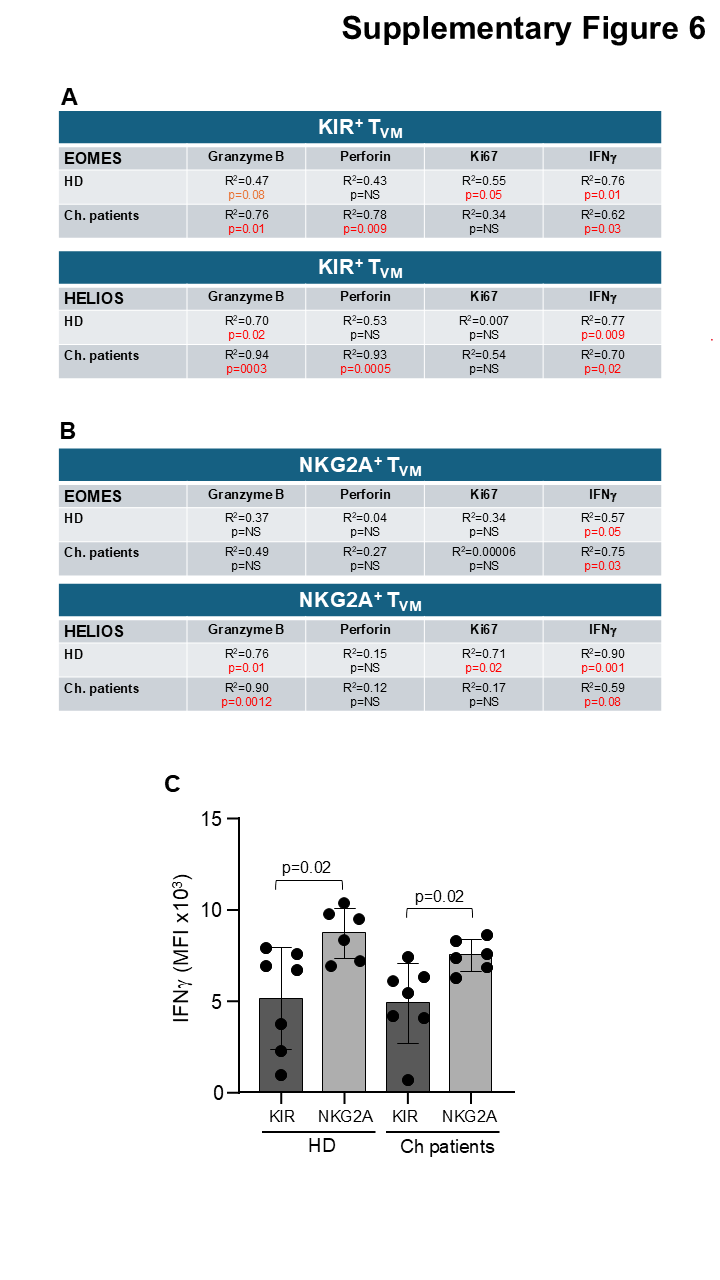

Supplement: Supplementary Figure 6 — Correlation of transcription factors and effector molecules expression in NKG2A+ and KIR+ TVM CD8+ T cells from healthy and T. cruzi-infected individuals. Correlations between the expression of the transcription factors Eomes or Helios and cytotoxic/activation markers (Granzyme B, Perforin, Ki67, IFNγ). R² and p-values were determined using simple linear regression analysis in (A) KIR+ and (B) NKG2A+ human TVM cells from healthy donors (HD) and Chagas patients (Ch patients). (C) Mean fluorescence intensity (MFI) of IFNγ+ cells in KIR+ and NKG2A+ cells between HD and Ch patients. Statistical significance was assessed by two-way ANOVA. [file Image6.tif]
